# Astroglial connexin 43 is a novel therapeutic target for chronic multiple sclerosis model

**DOI:** 10.1038/s41598-024-61508-2

**Published:** 2024-05-13

**Authors:** Ezgi Ozdemir Takase, Ryo Yamasaki, Satoshi Nagata, Mitsuru Watanabe, Katsuhisa Masaki, Hiroo Yamaguchi, Jun-ichi Kira, Hideyuki Takeuchi, Noriko Isobe

**Affiliations:** 1 Department of Neurology, Neurological Institute, Graduate School of Medical Sciences, Kyushu University, 3-1-1 Maidashi, Higashi-ku, Fukuoka, 812-8582 Japan; 2 School of Physical Therapy, Faculty of Rehabilitation, Reiwa Health Sciences University, Fukuoka, Japan; 3 Translational Neuroscience Center, Graduate School of Medicine, and School of Pharmacy at Fukuoka, International University of Health and Welfare, Ookawa, Japan; 4 Department of Neurology, Brain and Nerve Center, Fukuoka Central Hospital, Fukuoka, Japan; 5 Department of Neurology and Stroke Medicine, Graduate School of Medicine, Yokohama City University, 3-9 Fukuura, Kanazawa-ku, Yokohama, 236-0004 Japan; 6 Department of Neurology, Graduate School of Medicine, International University of Health and Welfare, Narita, Japan; 7 Center for Intractable Neurological Diseases and Dementia, International University of Health and Welfare Atami Hospital, Atami, Japan

**Keywords:** Experimental autoimmune encephalomyelitis, Connexin 43, Hemichannel, Gap junction, Multiple sclerosis, Microglia, Astroglia, Neuroimmunology, Astrocyte, Microglia, Multiple sclerosis, Mechanisms of disease, Experimental models of disease

## Abstract

In chronic stages of multiple sclerosis (MS) and its animal model, experimental autoimmune encephalitis (EAE), connexin (Cx)43 gap junction channel proteins are overexpressed because of astrogliosis. To elucidate the role of increased Cx43, the central nervous system (CNS)-permeable Cx blocker INI-0602 was therapeutically administered. C57BL6 mice with chronic EAE initiated by MOG_35-55_ received INI-0602 (40 mg/kg) or saline intraperitoneally every other day from days post-immunization (dpi) 17–50. Primary astroglia were employed to observe calcein efflux responses. In INI-0602-treated mice, EAE clinical signs improved significantly in the chronic phase, with reduced demyelination and decreased CD3^+^ T cells, Iba-1^+^ and F4/80^+^ microglia/macrophages, and C3^+^GFAP^+^ reactive astroglia infiltration in spinal cord lesions. Flow cytometry analysis of CD4^+^ T cells from CNS tissues revealed significantly reduced Th17 and Th17/Th1 cells (dpi 24) and Th1 cells (dpi 50). Multiplex array of cerebrospinal fluid showed significantly suppressed IL-6 and significantly increased IL-10 on dpi 24 in INI-0602-treated mice, and significantly suppressed IFN-γ and MCP-1 on dpi 50 in the same group. In vitro INI-0602 treatment inhibited ATP-induced calcium propagations of Cx43^+/+^ astroglial cells to similar levels of those of Cx43^−/−^ cells. Astroglial Cx43 hemichannels represent a novel therapeutic target for chronic EAE and MS.

## Introduction

Multiple sclerosis (MS) is an autoimmune demyelinating and neurodegenerative disease of the central nervous system (CNS). Various roles played by glial connexins (Cxs) have been reported in the pathogenicity of diseases characterized by neurodegeneration and demyelination^1,2^. Cxs comprise gap junctions (GJ) and hemichannels (HCs), and enable direct signaling between cells. GJs consist of apposed HCs on the plasma membranes of adjoining cells and are important for the direct propagation of calcium waves, exchange of metabolites, extracellular K^+^ buffering, and regulation of electrical synaptic transmission between neurons^3^. In the CNS, astroglia express Cx26, Cx30, and Cx43, whereas oligodendroglia express Cx29, Cx32, and Cx47. GJ coupling between astroglia is formed by homomeric and heteromeric combinations of Cx26 and Cx30, whereas Cx43 only forms homomeric channels. In astroglia–oligodendroglia GJ coupling, the predominant Cx combinations are Cx43–Cx47, Cx30–Cx32, and Cx26–Cx32. Under pathologic conditions, Cxs become HCs that present a high open probability state and secrete microglial chemoattractant factors, including glutamate and ATP, and inflammatory cytokines/chemokines extracellularly^4,5^. Inflammatory cytokines secreted from glial cell surface HCs promote demyelination^6,7^.

We previously reported the overexpression of astroglial Cx43 GJ channel proteins within the plaques of chronic MS (reflecting reactive astrogliosis) and, controversially, diminished oligodendroglial Cx47 expression^8^. This polarity between Cx expression levels in chronic-phase MS results in a relative elevation of Cx43 HCs. Recent publications have further illuminated the immunomodulatory functions of Cxs using an animal model of MS termed experimental autoimmune encephalitis (EAE)^9,10^. We recently reported that acute ablation of Cx47 in oligodendroglial *Cx47* inducible conditional knockout (icKO) mice resulted in a relative increase of Cx43 HCs, aggravated demyelination, and EAE signs^11^. Furthermore, glutamate aspartate transporter (GLAST)^+^ *Cx43* icKO mice, characterized by the loss of astroglia-specific Cx43 in brain gray matter, showed amelioration of EAE clinical signs, less demyelination in spinal cord lesions, and less immune cell infiltration and suppressed astroglial activation^12^.

We hypothesized that upregulated astroglial Cx43 in chronic EAE and MS lesions may aggravate neuroinflammation by inducing the inflammatory milieu. Here, we aimed to counteract the elevated HC formation resulting from Cx imbalances by pharmacologically inhibiting HC activity in both in vivo and in vitro models. A candidate HC blocker, carbenoxolone disodium (CBX), blocks GJ-HCs non-specifically^13^ but has limited medical use because of side effects associated with its mineralocorticoid-like actions^14^ and inability to pass through the intact blood–brain barrier (BBB)^15^. To overcome these limitations, we employed a pan-Cx blocker, INI-0602, which was developed to circumvent pseudoaldosteronism and enhance drug penetration into the CNS^15^. INI-0602 inhibits GJ/HC opening by binding to the outer loop of Cxs, leading to their irreversible internalization and effectively blocking GJ/HC. INI-0602 has successfully halted disease advancement in murine models of Alzheimer’s disease, Parkinson’s disease, amyotrophic lateral sclerosis, juvenile neuronal ceroid lipofuscinosis, neuropathic pain, and spinal cord injury^15–18^.

In this study, our objective was to clarify the function of excess Cx43 expression in EAE and MS using intraperitoneal (IP) injection of CNS-permeable INI-0602. Pharmacological blockade of Cx43 HCs with INI-0602 improved EAE clinical signs, slowed disease progression, and prevented excessive demyelination in the spinal cord. In vitro INI-0602 treatment halted glial activation by disrupting communication between neurotoxic astroglia, potentially by modifying astroglial calcium signaling. We speculate that INI-0602 is a promising therapeutic agent for chronic progressive MS, a condition for which few highly efficient drugs are available^19–21^.

## Results

### Therapeutic administration of INI-0602 attenuates chronic clinical EAE signs and suppresses demyelination

**Figure 1:**
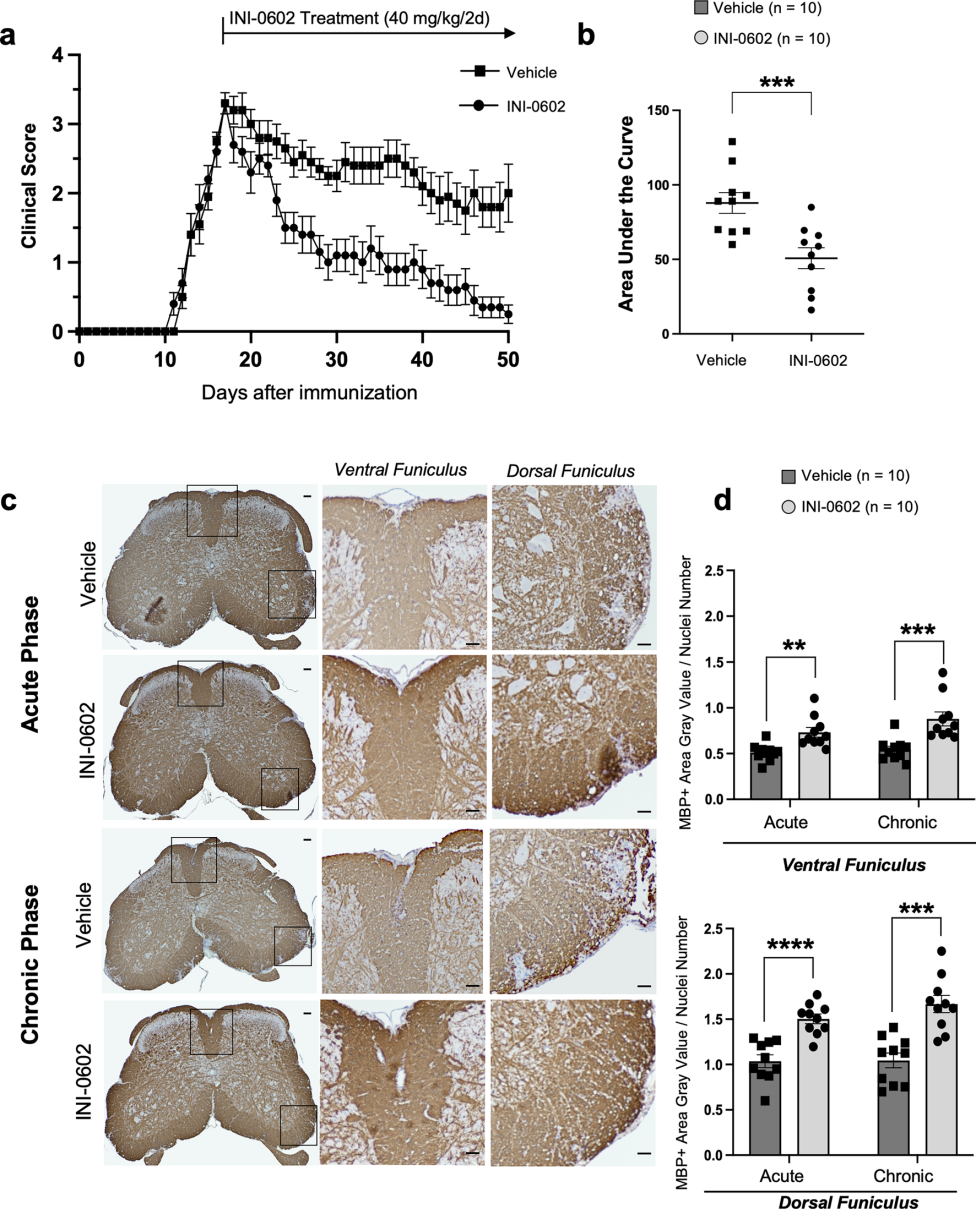
Amelioration of experimental autoimmune encephalomyelitis (EAE) scoring parameters and demyelination by INI-0602. (**a**) Daily EAE clinical scores (means ± SEM) are presented from the day of inoculation (day 0). B6 wild type (WT) mice were administered intraperitoneal saline (*n* = 10, vehicle) or INI-0602 (*n* = 10) on alternate days from dpi 17 to 50. (**b**) The severity of disease symptoms was examined by comparing area under the curve (AUC) values between dpi 17 and 50. Statistical data are presented as means ± SEM.* P*-values were computed using the Mann–Whitney test. ***P < 0.001. (**c**) Stained lumbar spinal cord sections with myelin basic protein (MBP) from INI-0602-treated and control (vehicle-treated) EAE mice at dpi 50. Enlarged views of the boxed sections are shown on the *right* (scale bars: 100 µm). (**d**) Myelin density of the ventral funiculus (VF) and dorsal funiculus (DF) in the lumbar spinal cord of EAE mice, determined by quantitative assessment of MBP immunostaining. Statistical data are presented as means ± SEM. *P*-values were computed using unpaired *t-*tests. **P < 0.01; ***P < 0.001; ****P < 0.0001.

First, we induced EAE in C57BL6 mice, treated them with INI-0602 or vehicle, and assessed disease severity over 50 days post-immunization (dpi). In the treatment group (administered INI-0602 from dpi 17), clinical signs of EAE were ameliorated compared with the vehicle group from the acute to chronic phases. INI-0602 induced a pronounced reduction of disease severity compared with the vehicle group, as evidenced by lower EAE clinical scores (area under the curve (AUC) of clinical scores at chronic phase (dpi 50), vehicle group vs. treatment group: 87.85 ± 3.643 vs. 50.830 ± 3.356, *P* = 0.0006; Fig. 1a,b). The acute phase (dpi 24) of the vehicle-treated EAE model was characterized by a drop in body weight caused by disease progression. As the disease continued into the chronic phase, body weight recovered in response to moderate disease activity. The body weights of mice treated with INI-0602 had a faster recovery than those of mice treated with vehicle (AUC, *P* = 0.0236; Supplementary Fig. S1).

Histopathological analysis performed on dpi 24 and 50 indicated a marked decrease in the degree of demyelination, as assessed by the myelin basic protein-immunopositive (MBP^+^) area, in the lumbar spinal cord of INI-0602-treated mice compared with vehicle-treated mice at both time points (ventral funiculus (VF): acute, *P* = 0.0018 and chronic, *P* = 0.0009; dorsal funiculus (DF): acute, *P* < 0.0001 and chronic, *P* = 0.0001; Fig. 1c,d).

As a preventive treatment protocol, we also administered INI-0602 or vehicle to mice beginning on the day of immunization with myelin oligodendrocyte glycoprotein (MOG)_35–55,_ when no clinical symptoms were observed, and continuing until dpi 24. Although symptom progression from onset to peak was comparable to that of the control group, there was a significant improvement in symptom alleviation following the peak (*P* = 0.0455; Supplementary Fig. S2a,b). However, because our study focused on the development of interventional therapeutic drugs, we did not perform full immunohistological investigations of this preventive therapy.

### Therapeutic administration of INI-0602 attenuates acute and chronic EAE by suppressing microglial activation and inflammatory cell infiltration

**Figure 2:**
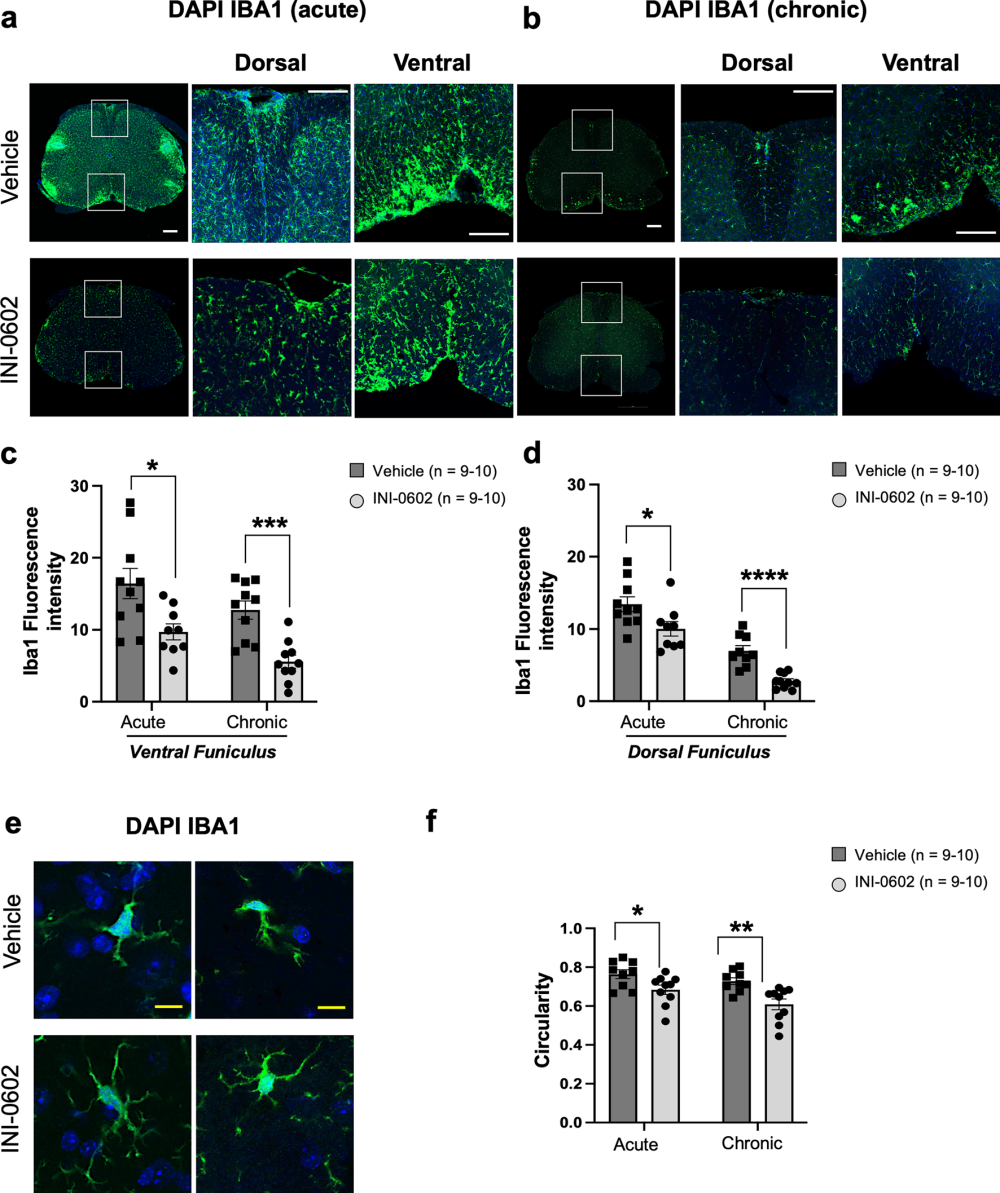
Inhibition of microglial activation by INI-0602 treatment. (**a**, **b**) INI-0602 treatment diminished the elevation of Iba-1 levels in the lumbar spinal cord of EAE mice at acute (dpi 24) (**a**) and chronic (dpi 50) (**b**) phases. Scale bar: 200 µm. (**c**, **d**) Areas of Iba-1^+^ (green) cells evaluated on dpi 24 and 50 in the VF (**c**) and DF (**d**). (**e**) Immunofluorescent image, captured at 40 × magnification, displaying Iba-1^+^ cells from both acute and chronic EAE lesions in the lumbar spinal cord (yellow scale bar: 10 µm). (**f**) Iba-1^+^ cell circularity quantified at dpi 24 (acute) and 50 (chronic). Cell nuclei were counterstained with DAPI (blue). Statistical data are presented as means ± SEM. *P*-values were computed using unpaired *t-*tests. *P < 0.05; **P < 0.01; ***P < 0.001; ****P < 0.0001.

We assessed glial activation and expanded CNS entry of inflammatory cells after EAE induction, and assessed the influence of INI-0602 on this process in both acute and chronic EAE. Allograft inflammatory factor 1 (Iba-1)^+^ cells in the lumbar spinal cord were more abundant in vehicle-treated mice than in INI-0602-treated mice at both acute and chronic phases (VF: acute, *P* = 0.0146 and chronic, *P* = 0.0002; DF: acute, *P* = 0.0299 and chronic, *P* < 0.0001; Fig. 2a–d).

Vehicle-treated mice exhibited higher values of Iba-1^+^ cell circularity and showed less-ramified morphology compared with those in INI-0602-treated mice, indicating that INI-0602 treatment leads to reduced microglial activation (acute, *P* = 0.0288; chronic, *P* = 0.0064; Fig. 2e,f).

**Figure 3:**
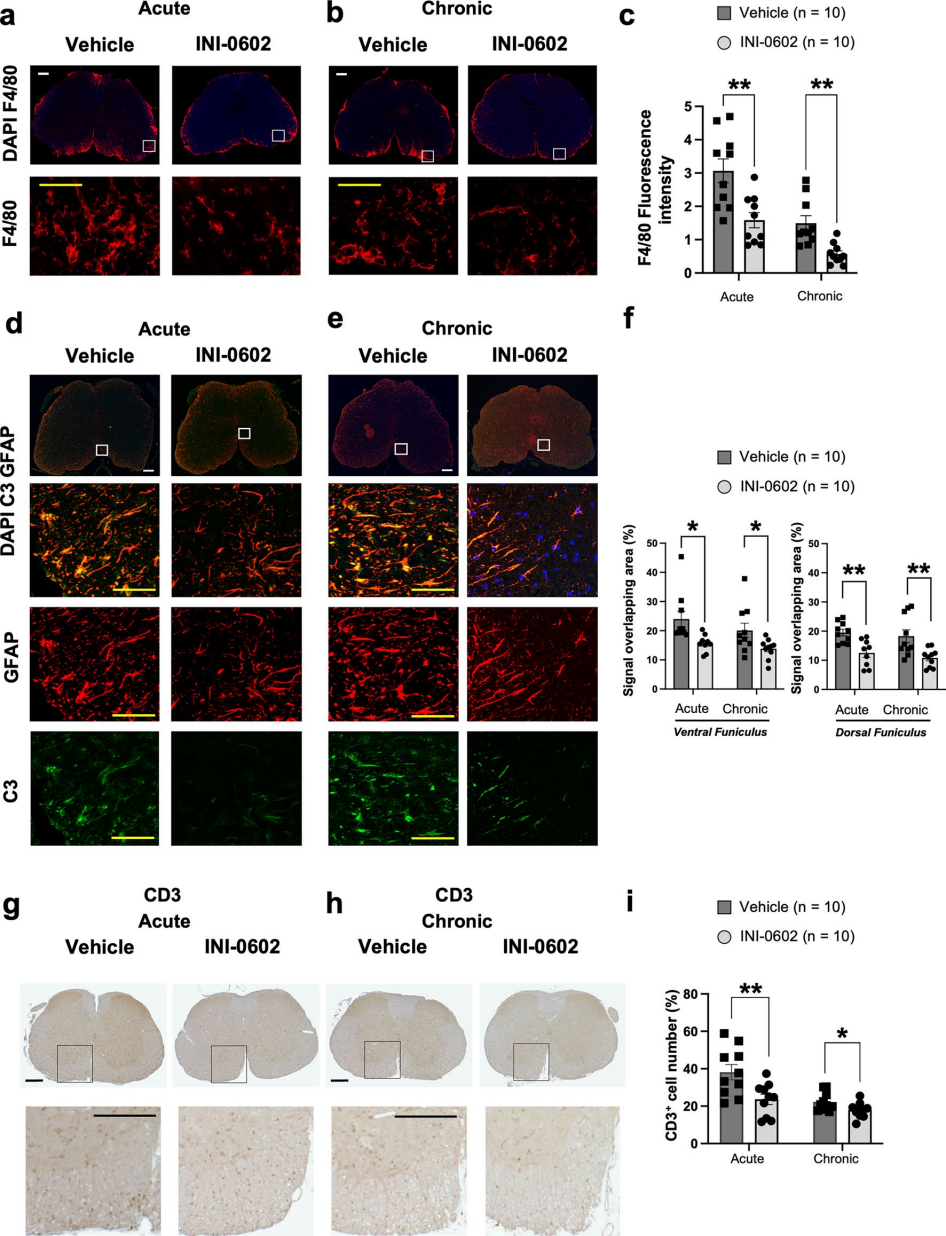
Therapeutic administration of INI-0602 attenuates acute and chronic EAE by inhibiting inflammatory cell infiltration. (**a**–**c**) Fluorescent intensity of F4/80^+^ (red) cells evaluated at acute (dpi 24) (**a**) and chronic (dpi 50) (**b**) phases (scale bar: 100 µm). Enlarged views of the boxed sections are shown at the *bottom* (yellow scale bar: 25 µm). (**d**–**f**) Overlapping areas of GFAP^+^ (red) and C3^+^ (green) cells quantified at acute (dpi 24) (**d**) and chronic (dpi 50) (**e**) phases (scale bar: 100 µm). Enlarged views of the boxed sections are shown at the *bottom* (yellow scale bar: 25 µm). (**g**–**i**) Number of CD3^+^ cells quantified at acute (dpi 24) (**g**) and chronic (dpi 50) (**h**) phases (scale bar: 100 µm). Enlarged views of the boxed sections are shown at the *bottom* (scale bar: 100 µm). Statistical data are presented as means ± SEM. *P*-values were computed using unpaired *t*-tests. *P < 0.05; **P < 0.01.

Next, assessment of F4/80^+^ cells revealed expanded infiltration in the white matter of the lumbar spinal cord of vehicle-treated mice compared with INI-0602-treated mice at the chronic phase (acute, *P* = 0.0026; chronic, *P* = 0.0013; Fig. 3a–c).

Inflammatory activation of C3^+^ glial fibrillary acidic protein (GFAP)^+^ astroglia (VF: acute, *P* = 0.0123 and chronic, *P* = 0.0348; DF: acute, *P* = 0.0016 and chronic, *P* = 0.0068; Fig. 3d–f) and infiltration of cluster of differentiation (CD)3^+^ T cells (acute, *P* = 0.0092 and chronic, *P* = 0.0448; Fig. 3g–i) in lumbar spinal cord lesions were significantly suppressed by INI-0602 treatment at both acute and chronic phases.

These findings indicate that in INI-0602-treated EAE mice, cellular inflammatory activation and infiltration into the spinal cord are less evident compared with that observed in vehicle-treated mice.

### EAE-induced synapse loss in lamina IX of the lumbar spinal cord is recovered by INI-0602 treatment

**Figure 4:**
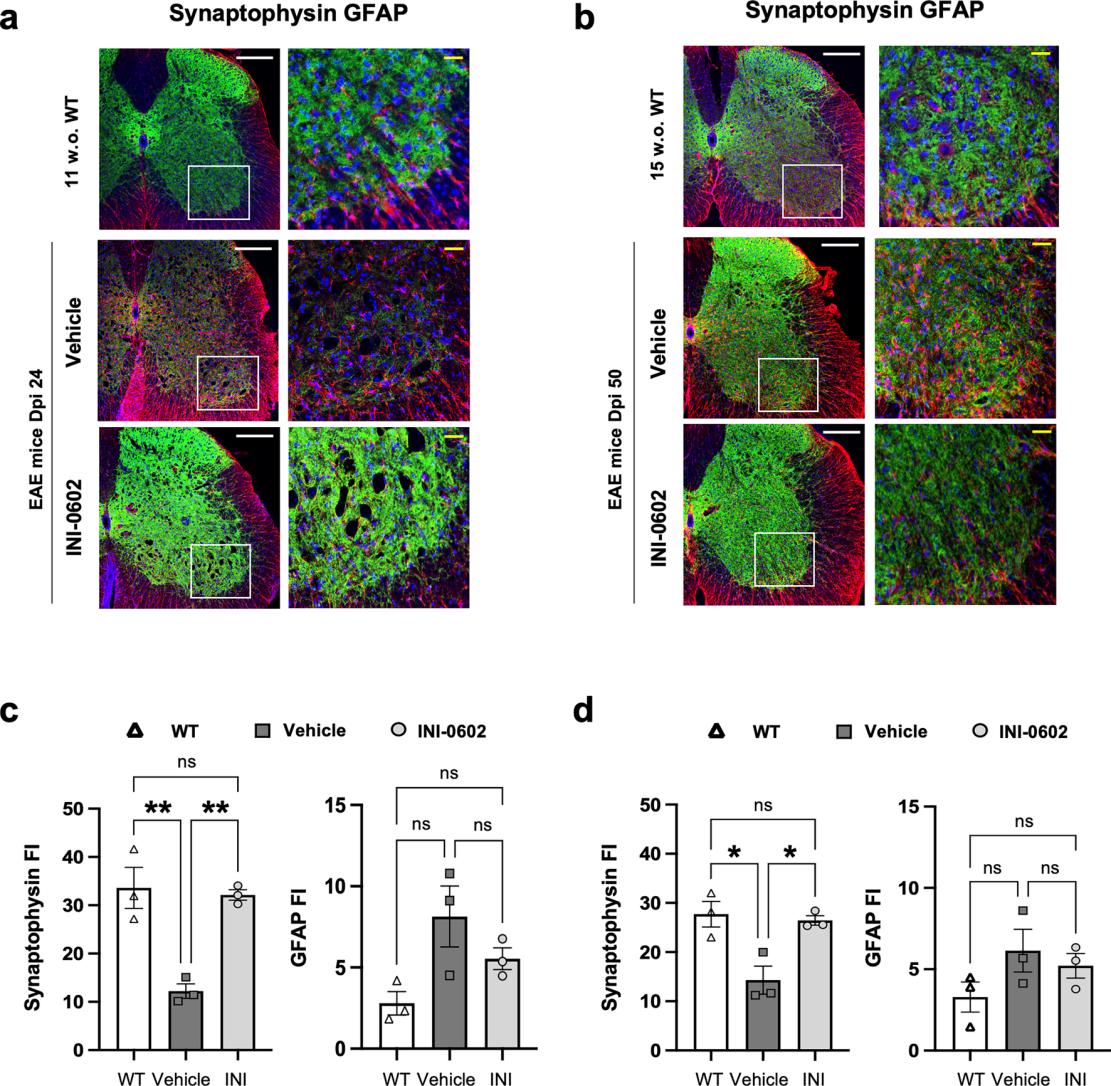
Comparison of areas immunopositive for the synaptic marker synaptophysin and GFAP between WT and EAE mice before and after INI-0602 treatment. (a,b) Immunofluorescent staining of GFAP (red) and synaptophysin (green) from WT littermates (11 w.o.) and vehicle- or INI-0602 treated EAE mice on dpi 24 (**a**), and WT littermates (15 w.o.) and vehicle- or INI-0602 treated EAE mice on dpi 50 (**b**) (scale bar: 100 µm). Enlarged views of the boxed sections are shown on the *right* (yellow scale bar: 50 µm). (**c**, **d**) Assessment of the fluorescent intensity (FI) (in arbitrary units) of synaptophysin- and GFAP-stained areas within the lamina IX region of the lumbar spinal cord of WT (11 w.o.), vehicle-treated, and INI-0602-treated EAE mice on dpi 24 (*n* = 3 per group) (**c**), and of WT (15 w.o.), vehicle-treated, and INI-0602-treated EAE mice on dpi 50 (*n* = 3 per group). *P*-values were computed using one-way ANOVA with Bonferroni post hoc tests. *P < 0.05; **P < 0.01.

To investigate the association between synaptic marker expression and GFAP expression in lamina IX of the lumbar spinal cord before and after INI-0602 treatment, we performed immunofluorescent staining of the synaptic vesicle integral membrane protein marker synaptophysin and the astroglial marker GFAP. Synaptophysin immunoreactivity in lamina IX of the lumbar spinal cord of vehicle-treated EAE mice on dpi 24 was significantly reduced compared with that of their wild-type (WT) littermates at 11 weeks old (w.o.) (11 w.o. WT mice vs. vehicle group: *P* = 0.0040; Fig. 4a,c), whereas GFAP expression showed a tendency to increase (11 w.o. WT mice vs. vehicle group: *P* = 0.0640; Fig. 4a,c). INI-0602 treatment of EAE mice from dpi 17 to 24 resulted in a significant increase in synaptophysin expression compared with vehicle (vehicle group vs. treatment group: *P* = 0.0057; Fig. 4a,c), rising to similar levels as in their 11 w.o. WT littermates (11 w.o. WT mice vs. treatment group: *P* > 0.9999; Fig. 2a,c). GFAP expression was not altered by INI-0602 treatment compared with vehicle-treated mice or their WT littermates during the acute phase (vehicle group vs. treatment group: *P* = 0.5490, 11 w.o. WT mice vs. treatment group: *P* = 0.4908; Fig. 2b,d). Synaptophysin immunoreactivity was reduced in vehicle-treated EAE mice on dpi 50 compared with WT littermates at 15 w.o. (15 w.o. WT mice vs. vehicle group: *P* = 0.0186; Fig. 4b,d), whereas GFAP expression was unchanged (15 w.o. WT mice vs. vehicle group: *P* = 0.2921; Fig. 4b,d). INI-0602 treatment from dpi 17 to 50 significantly increased synaptophysin expression compared with vehicle-treated mice (vehicle group vs. treatment group: *P* = 0.0292; Fig. 4a,c), to levels similar to those of their WT littermates (15 w.o. WT mice vs. treatment group: *P* > 0.9999; Fig. 4a,c). GFAP levels were unchanged by INI-0602 treatment compared with vehicle-treated mice or their WT littermates during the chronic phase (vehicle group vs. treatment group: *P* > 0.9999, 15 w.o. WT mice vs. treatment group: *P* = 0.6988; Fig. 4b,d).

### INI-0602 treatment inhibits infiltration of helper T(Th)17 and Th1 cell migration into the CNS

**Figure 5:**
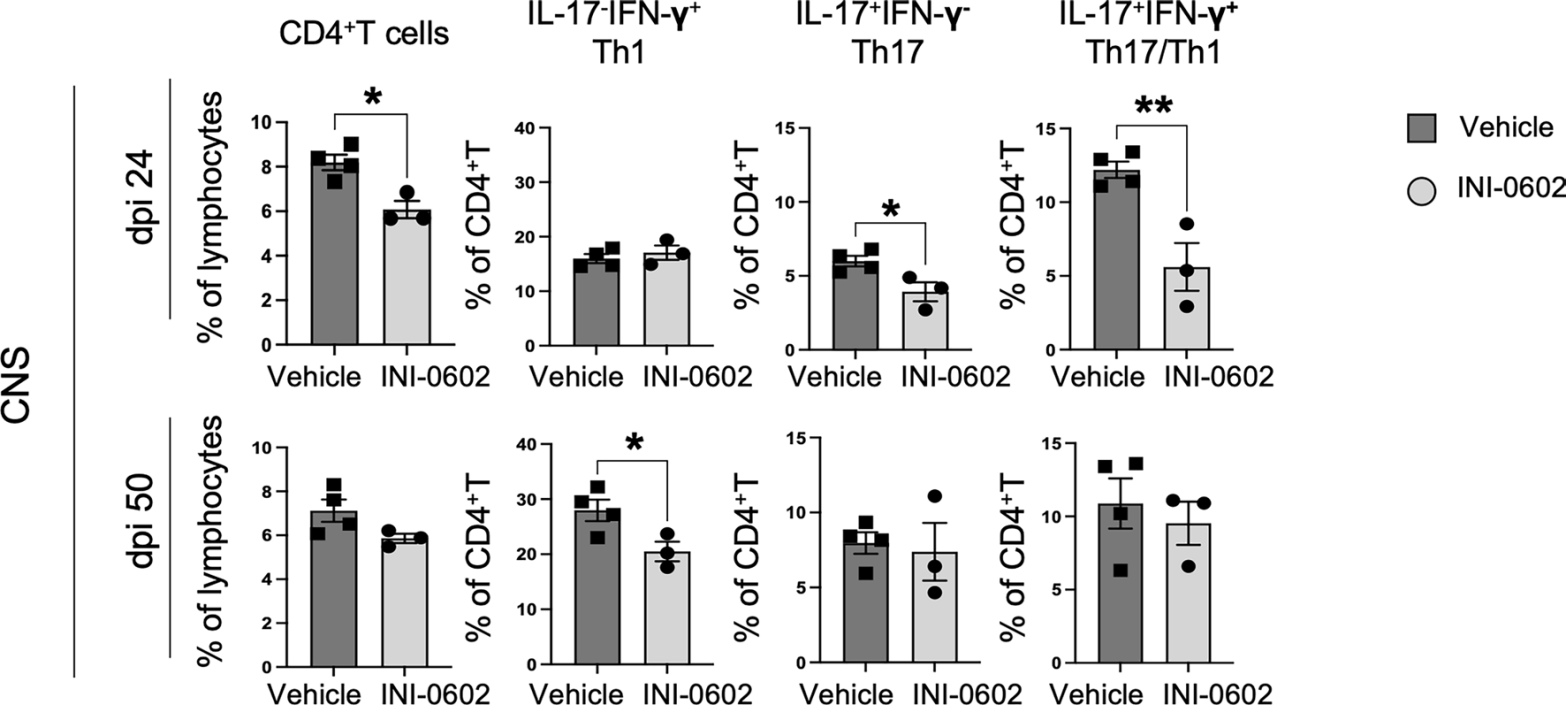
Flow cytometry analysis of T cells isolated from CNS tissues of acute (dpi 24) and chronic (dpi 50) EAE mice for intracellular cytokine assays and the quantification of individual cell populations. Quantitative analysis of IL-17^−^IFN-γ^+^CD4^+^ (helper T(Th)1), IL-17^+^IFN-γ^−^CD4^+^ (Th17), and IL-17^+^IFN-γ^+^CD4^+^ (Th17/Th1) cell percentages in CD4^+^ T cells from the spinal cord of INI-0602- and vehicle-treated EAE mice on dpi 24 and 50. Statistical data are presented as means ± SEM (*n* = 3–4 mice per group). *P*-values were computed using unpaired *t*-tests. *P < 0.05; **P < 0.01.

Next, to elucidate the characteristics of T cells infiltrated into lesions, we assessed interferon (IFN)-γ-yielding Th1 cells and interleukin (IL)-17A-yielding Th17 cells using flow cytometry of CD4^+^ T cells (Supplementary Fig. S4 displays representative flow cytometry plots illustrating the gating scheme) obtained from the spinal cord of acute (dpi 24) and chronic (dpi 50) EAE mice. Administration of INI-0602 from the peak of EAE (dpi 17) to the acute phase significantly decreased CD4^+^ T cells, as well as IL-17A^+^IFN-γ^−^ Th17 and IL-17A^+^IFN-γ^+^ Th17/Th1 cell percentages in CD4^+^ T cells that were obtained from the spinal cord (*P* = 0.0100 for CD4^+^ T cells, *P* = 0.0290 for IL-17A^+^IFN-γ^−^ Th17 cells, *P* = 0.0073 for IL-17A^+^IFN-γ^+^ Th17/Th1 cells; Fig. 5). INI-0602 administration from the peak of EAE to the chronic phase significantly decreased IL-17A^−^IFN-γ^+^ Th1 cell percentages (*P* = 0.0413; Fig. 5). FoxP3^+^ regulatory T (Treg) cell percentages were not changed in acute and chronic EAE (acute, *P* = 0.3523; chronic, *P* = 0.6886; Supplementary Fig. S4r,s).

Additionally, we assessed splenic Th1, Th17, Th17/Th1, and FoxP3^+^ Treg cells by flow cytometry (Supplementary Fig. S5 displays representative flow cytometry plots illustrating the gating scheme) among cells obtained from splenocyte tissue. Treg (acute, *P* = 0.9448; chronic, *P* = 0.2397; Supplementary Fig. S5r,s) and Th (acute, *P* = 0.1669 for CD4^+^ T cells*, P* = 0.8855 for IL-17A^−^IFN-γ^+^ Th1, *P* = 0.9537 for IL-17A^+^IFN-γ^−^ Th17, *P* = 0.8337 for IL-17A^+^IFN-γ^+^ Th17/Th1; chronic, *P* = 0.8066 for CD4^+^ T cells, *P* = 0.8159 for IL-17A^−^IFN-γ^+^ Th1, *P* = 0.5242 for IL-17A^+^IFN-γ^−^ Th17, *P* = 0.4492 for IL-17A^+^IFN-γ^+^ Th17/Th1; Supplementary Fig. S5r,s) cells exhibited no substantial differences between the treatment and control groups. In summary, therapeutic administration of INI-0602 appears to mitigate the severity of acute EAE (by inhibiting Th17 and Th17/Th1) and chronic EAE (by restraining Th1 cell infiltration into CNS tissues).

### MOG-specific T cell responses show no discrepancies between vehicle- and INI-0602-treated mice

Splenocytes obtained from mice treated with either vehicle or INI-0602 during the acute (dpi 24) and chronic (dpi 50) stages of EAE were stimulated with varying concentrations of MOG_35–55_. No notable differences in MOG-specific proliferation were observed between the two groups at either time point (Fig. S6c,d).

### Distribution of Cx43 and Cx47 is altered during EAE in the lumbar spinal cord and INI-0602 reduces Cx43 expression in acute and chronic EAE

**Figure 6:**
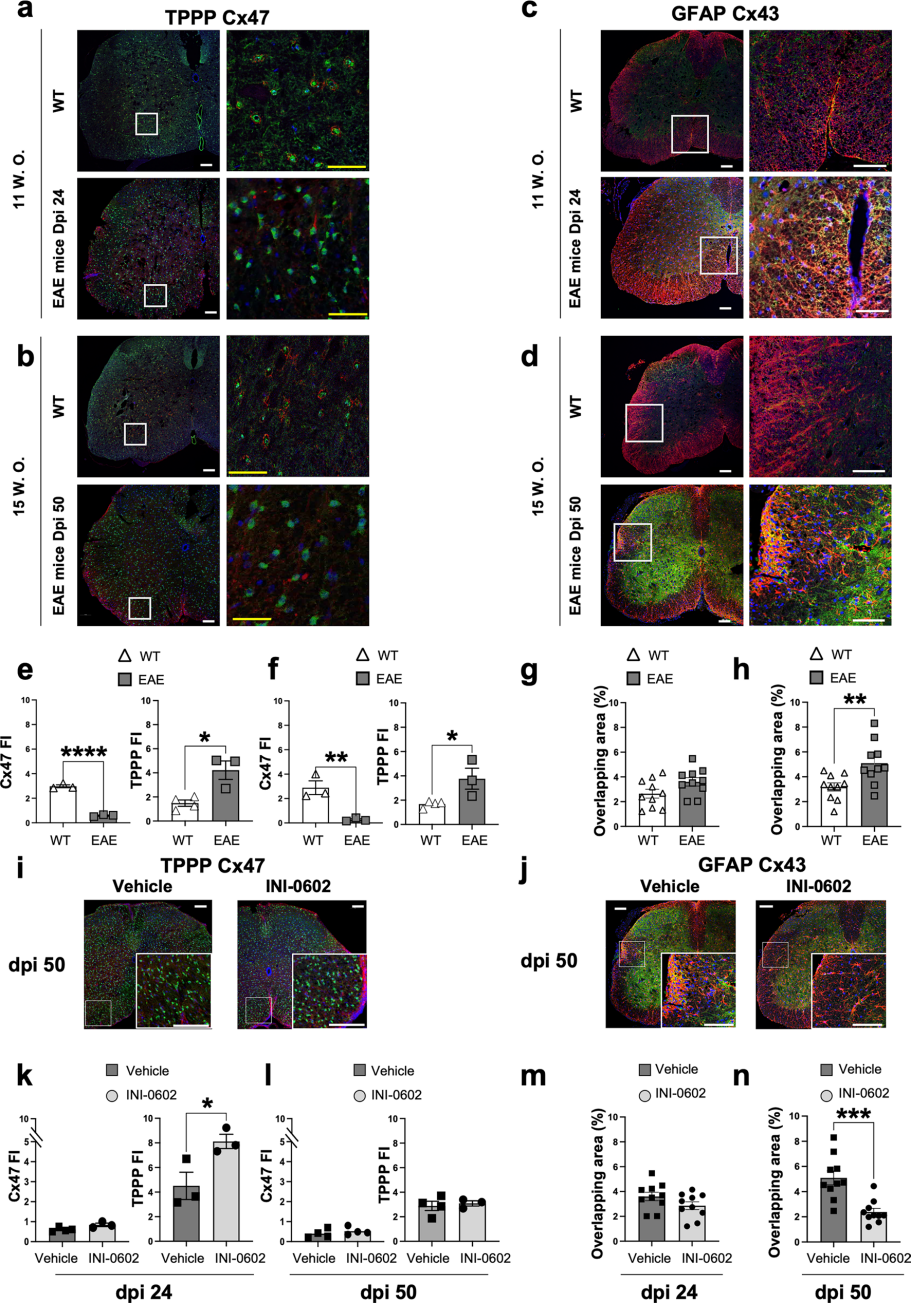
Distribution of Cx43/Cx47 throughout the EAE mouse and alterations of Cx43/Cx47 in the lumbar spinal cord after INI-0602 treatment. (a,b) Immunofluorescent staining of Cx47 (red) and the oligodendroglia marker TPPP (green) from dpi 24 EAE mice and their WT littermates (11 w.o.) (**a**), and from dpi 50 EAE mice and their WT littermates (15 w.o.) (**b**) (scale bar: 100 µm). Enlarged views of the boxed sections are shown on the *right* (yellow scale bar: 50 µm). (**c**, **d**) Immunofluorescent staining of Cx43 (green) and the astroglial marker GFAP (red) from dpi 24 (**c**) and 50 (**d**) EAE mice (scale bar: 100 µm). (**e**, **f**) Assessment of the fluorescent intensity (FI) (in arbitrary units) of the Cx47^+^ and TPPP^+^ area of dpi 24 (*n* = 3–4 per group) (**e**) and dpi 50 (*n* = 3–4 per group) (f) EAE mice. (**g**, **h**) Overlapping area of Cx43^+^GFAP^+^ staining from dpi 24 (*n* = 10 per group) (**g**) and dpi 50 (*n* = 10 per group) (**h**) EAE mice. (**i**, **j**) Immunofluorescent staining of Cx47 and TPPP (**i**) and GFAP and Cx43 (**j**) after INI-0602 treatment of EAE mice until dpi 50 (scale bar: 100 µm). (**k**, **l**) Statistical evaluation of Cx47^+^ or TPPP^+^ cell area between vehicle- and INI-0602-treated mice on dpi 24 (*n* = 3–4 per group) (**k**) and dpi 50 (*n* = 3–4 per group). (**m**, **n**) Statistical evaluation of the overlapping area of Cx43 and GFAP staining between vehicle- and INI-0602-treated mice on dpi 24 (*n* = 10 per group) (**m**) and dpi 50 (*n* = 10 per group) (**n**). *P*-values were computed using unpaired *t-*tests. *P < 0.05; **P < 0.01; ***P < 0.001; ****P < 0.0001.

Next, we compared oligodendroglial-Cx47 and astroglial-Cx43 expression levels between EAE mice and their healthy littermate counterparts to assess whether Cx expression patterns in EAE mimic those in MS patients. We also examined the effect of INI-0602 treatment on Cx expression in EAE mice. Quantification of immunofluorescent staining confirmed that Cx47 expression significantly diminished in the spinal cord white matter during the development of acute EAE (11 w.o. WT mice vs. dpi 24 EAE mice: *P* < 0.0001; Fig. 6a,e) and remained at similar levels to those in the chronic phase of the disease (15 w.o. WT mice vs. dpi 50 mice: *P* = 0.0099; Fig. 6b,f). Moreover, astroglial Cx43 expression showed a tendency toward overexpression in acute EAE (11 w.o. WT mice vs. dpi 24 EAE mice: *P* = 0.0680; Fig. 6c,g) and significantly increased in chronic EAE (15 w.o. WT mice vs. dpi 50 EAE mice: *P* = 0.0076; Fig. 6d,h). In vivo INI-0602 treatment of EAE mice from the peak of EAE (dpi 17) to the acute EAE phase (dpi 24) resulted in no significant changes in astroglial Cx43 expression (*P* = 0.1247; Fig. 6j,m), whereas continuation of INI-0602 treatment until the chronic EAE phase (dpi 50) resulted in significantly decreased astroglial Cx43 expression (*P* = 0.0004; Fig. 6j,n). Furthermore, Cx47 expression was not restored after treatment and maintained low expression levels in both acute and chronic EAE (acute, *P* = 0.0628, chronic, *P* = 0.4343; Fig. 6i,k,l). Quantification of tubulin polymerization promoting protein (TPPP)^+^ oligodendroglia revealed that the immunopositive cell area was significantly higher in dpi 24 EAE mice than in 11 w.o. WT mice (*P* = 0.0120; Fig. 6e) and in dpi 50 EAE mice than in 15 w.o. WT mice (*P* = 0.0370; Fig. 6f). Moreover, INI-0602 treatment of EAE mice from dpi 17 to 24 resulted in a significant increase in TPPP expression compared with vehicle (*P* = 0.0444; Fig. 6k); however, INI-0602 treatment of EAE mice from dpi 17 to 50 resulted in no significant change in expression levels of TPPP (*P* = 0.6945; Fig. 6l). These findings indicate that INI-0602 treatment of EAE mice inhibits the overexpression of astroglial Cx43 in the chronic phase and may regulate the glial syncytium.

### In vitro administration of INI-0602 changes astroglial phenotypes from pro-inflammatory to anti-inflammatory

**Figure 7:**
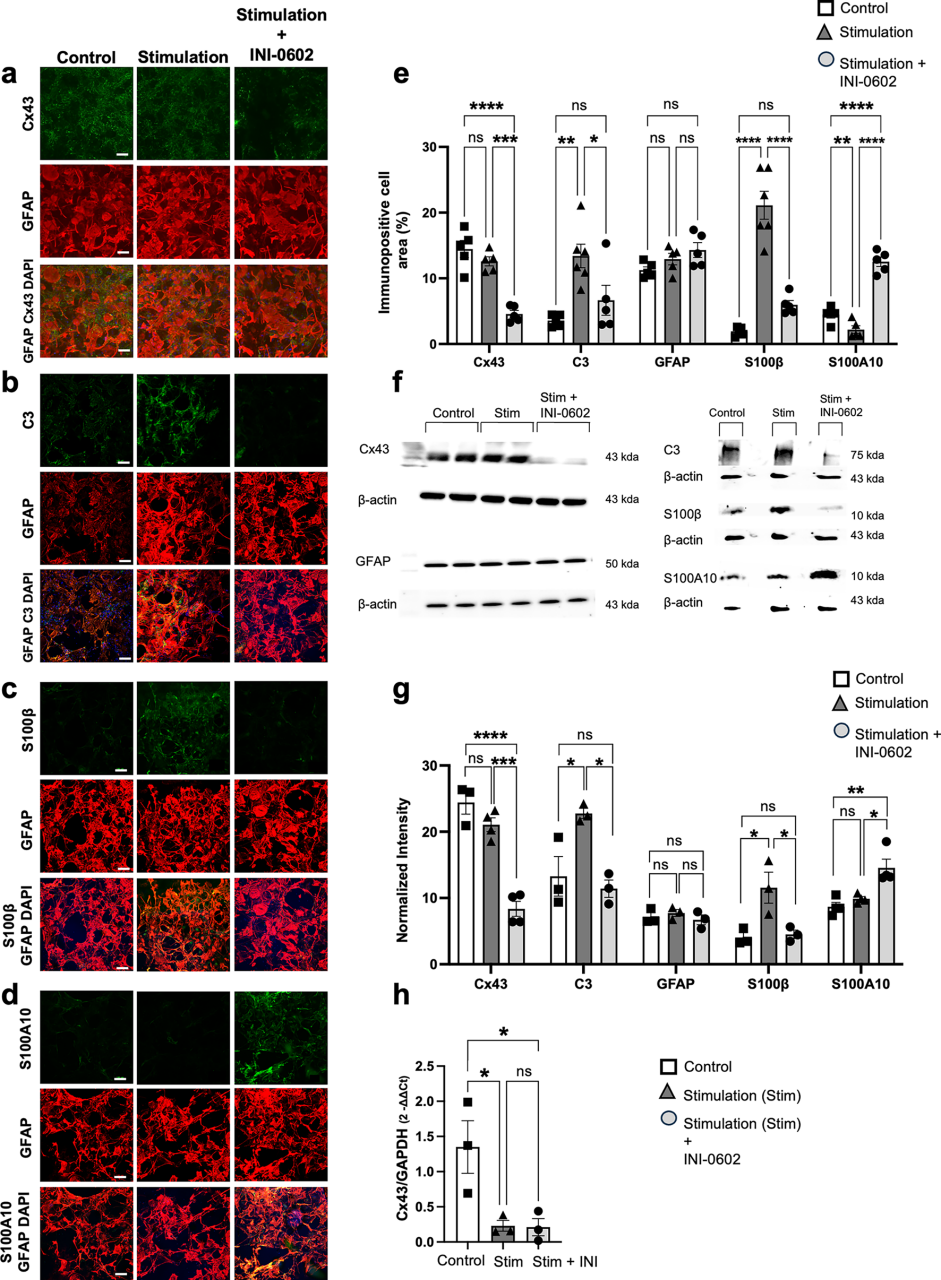
In vitro INI-0602 treatment prevents pro-inflammatory activation of astroglia. (a–d) Immunostaining with Cx43 (green)/GFAP (red) (**a**), C3 (green)/GFAP (red) (**b**), S100β (green)/GFAP (red) (**c**), S100A10 (green)/GFAP (red) (**d**) and DAPI (blue) in fixed cells (scale bars: 100 µm). (**e**) Quantification of the Cx43^+^, C3^+^, GFAP^+^, S100β^+^, and S100A10^+^ cell areas. *P*-values were computed using one-way ANOVA with Bonferroni post hoc tests (n = 4–5 per group). (**f**, **g**) Protein levels of Cx43, C3, GFAP, S100β, and S100A10 were measured using western blotting; β-actin served as the control for data normalization. Data are presented as means ± SEM (n = 3–4 per group) and were compared with the control using one-way ANOVA with Bonferroni post hoc tests. (**h**) Quantitative real-time RT-PCR bar graphs showing the fold change (2^−ΔΔCt^) in *Cx43* (*Gja1*) mRNA levels (levels were normalized to *Gapdh* mRNA as an internal control) with a reference of 1 for *Cx43/Gapdh* of the control. Statistical data are presented as means ± SEM (*n* = 3 per group). *P*-values were computed using one-way ANOVA with Bonferroni post hoc tests. *P < 0.05; **P < 0.01; ***P < 0.001; ****P < 0.0001; ns: non-significant. Full image blots are provided in Supplementary Fig. S8a–e.

After demonstrating that in vivo INI-0602 treatment reduced the expression of reactive astroglial markers in EAE mice, we explored the direct impact of Cx blockade on astroglia that were induced to a reactive pro-inflammatory state in vitro. We performed immunocytochemical analysis and western blotting of primary astroglial cell lysates after treatment with stimulatory mixture [SM; including IL-1α, tumor necrosis factor alpha (TNF-α), and complement component 1, q subcomponent (C1q)], with SM ± in vitro INI-0602, or without any treatment (for the control group). Immunocytochemical analysis showed that the Cx43^+^ areas of astroglia were comparable between control and stimulated cells (*P* = 0.3429; Fig. 7a,e); meanwhile, INI-0602-treated cells showed Cx43^+^ area reduction (stimulated vs. INI-0602-treated cells, *P* = 0.0001; control vs. INI-0602-treated cells, *P* < 0.0001; Fig. 7a,e). Western blot analysis of Cx43 protein levels showed similar results between control and stimulated cells (*P* = 0.3206; Fig. 7f,g), and between stimulated and INI-0602-treated cells (stimulated vs. INI-0602-treated cells, *P* = 0.0002; control vs. INI-0602-treated cells, *P* < 0.0001; Fig. 7f,g).

Immunocytochemical analysis showed that GFAP and markers positive areas, which are upregulated in inflammatory astroglia (C3 and S100β) were increased with stimulation (control vs. stimulated cells, C3: *P* < 0.0032; GFAP: *P* = 0.3875; S100β: *P* < 0.0001; Fig. 7b,c,e), and elevated protein levels of these markers were observed in western blot (C3: *P* = 0.0403; GFAP: *P* = 0.9999; S100β: *P* = 0.0326; Fig. 7f,g). Conversely, cells receiving SM treatment simultaneously with in vitro INI-0602 showed decreased pro-inflammatory markers positive area of activated astroglia compared with vehicle-treated cells (stimulated vs. INI-0602-treated cells, C3: *P* = 0.0359; GFAP: *P* = 0.5267; S100β: *P* < 0.0001; control vs. INI-0602-treated cells, C3: *P* = 0.4516; GFAP: *P* = 0.0698; S100β: *P* = 0.1780; Fig. 7b,c,e), along with decreased protein levels of these markers (stimulated vs. INI-0602-treated cells, C3: *P* = 0.0182; GFAP, *P* = 0.8819; S100β, *P* = 0.0427; control vs. INI-0602-treated cells, C3: *P* = 0.9999; GFAP: *P* = 0.9999; S100β: *P* = 0.9999; Fig. 7f,g). We also found that the positive area of a marker upregulated in anti-inflammatory astroglia (S100A10) was not significantly different between stimulated and control cells (*P* = 0.0554; Fig. 7d,e), and similar outcomes were noted with S100A10 protein levels (*P* = 0.6737; Fig. 7f,g). As anticipated, cells receiving INI-0602 treatment showed significantly increased S100A10^+^ cell area (stimulated vs. INI-0602-treated cells, *P* < 0.0001; control vs. INI-0602-treated cells, *P* < 0.0001; Fig. 7d,e) compared with vehicle-treated cells; this finding was confirmed by western blot analysis (stimulated vs. INI-0602-treated cells, *P* = 0.0456; control vs. INI-0602-treated cells, *P* = 0.0060; Fig. 7f,g).

These results suggest that Cx blockade prevents pro-inflammatory activation and promotes the anti-inflammatory reversion of astroglia under stimulatory conditions.

Next, we evaluated *Cx43* mRNA levels in astroglial cell cultures before and after treatment with SM ± in vitro INI-0602. Assessment with quantitative real-time reverse transcriptase (RT) polymerase chain reaction (PCR) showed that stimulation of astroglia by SM corresponded with downregulation of *Cx43* mRNA expression (*P* = 0.0311; Fig. 7h). By contrast, concurrent Cx blockade by INI-0602 and SM treatment resulted in no change in *Cx43* mRNA levels compared with cells that received SM stimulation only (*P* = 0.9999; Fig. 7h); however, the levels were decreased compared with control cells (*P* = 0.0392; Fig. 7h).

### Cerebrospinal fluid (CSF) cytokine levels are decreased in INI-0602-treated mice at the chronic phase of EAE

**Figure 8:**
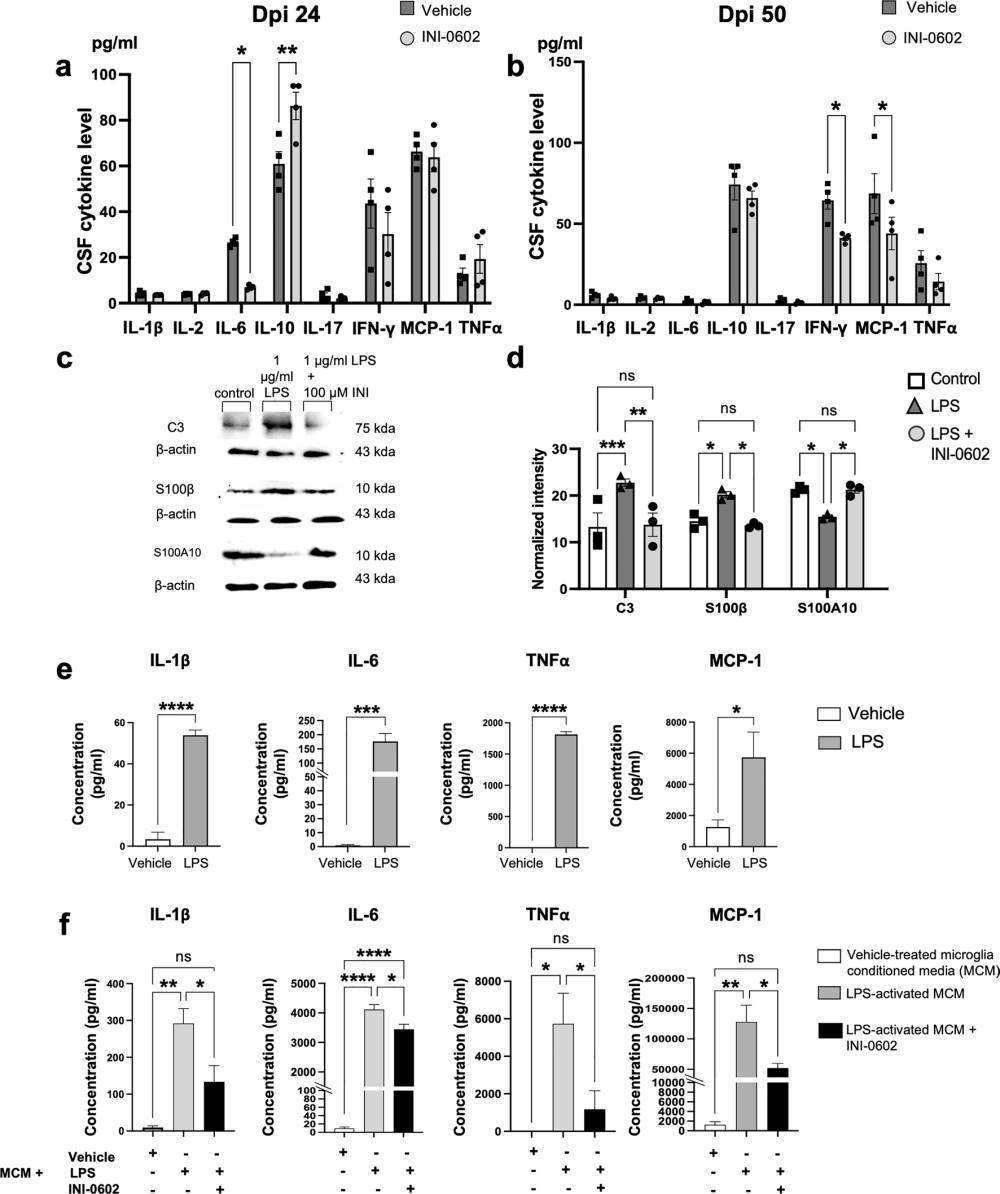
Inflammatory cytokine/chemokine levels in mouse CSF and astroglial cell culture media after INI-0602 treatment. (**a**, **b**) CSF cytokine levels of mice at the acute (dpi 24) (**a**) and chronic (dpi 50) (**b**) phases of EAE. Each bar corresponds to the mean cytokine/chemokine level ± SEM (*n* = 4). *P*-values were computed using unpaired *t*-tests. (**c**, **d**) Protein levels of pro-inflammatory astroglial markers (C3 and S100β) and an anti-inflammatory astroglial marker (S100A10) were measured in collected astroglial cell lysates using western blotting; β-actin served as the control for data normalization, with a reference of 1 for protein/β-actin of the control. Statistical data are presented as means ± SEM (*n* = 4). *P*-values were computed using one-way ANOVA with Bonferroni post hoc tests. *P < 0.05; **P < 0.01; ***P < 0.001; ns: non-significant. (**e**) Cytokine/chemokine levels in microglial culture media with or without LPS. Statistical data are presented as means ± SEM (*n* = 4). *P*-values were computed using unpaired *t*-tests. *P < 0.05; ***P < 0.001; ****P < 0.0001. (**f**) Cytokine/chemokine levels in astroglial culture media pretreated with vehicle-treated MCM, LPS-activated MCM, or LPS-activated MCM + INI-0602. Each bar corresponds to the mean cytokine/chemokine level ± SEM (*n* = 4).* P*-values were computed using one-way ANOVA with Bonferroni post hoc tests. *P < 0.05; **P < 0.01; ****P < 0.0001; ns: non-significant. Full image blots are presented in Supplementary Fig. S9a–c.

We collected CSF and serum from EAE mice in the treatment group (dpi 24: *n* = 4, dpi 50: *n* = 4) and control group (dpi 24: *n* = 4, dpi 50: *n* = 4) to measure cytokines/chemokines via multiplex array assay. Unpaired *t-*tests showed significant suppression of IL-6 and significant increase of IL-10 on dpi 24 (IL-1β: *P* = 0.3196; IL-2: *P* = 0.5249; IL-6: *P* = 0.0001; IL-10: *P* = 0.0196; IL-17: *P* = 0.3520; IFN-γ: *P* = 0.3850; C–C motif chemokine 2 (MCP-1): *P* = 0.7302; TNF-α: *P* = 0.3767; Fig. 8a) and significant suppression of IFN-γ and MCP-1 on dpi 50 (IL-1β: *P* = 0.0956; IL-2: *P* = 0.6142; IL-6: *P* = 0.3445; IL-10: *P* = 0.4522; IL-17: *P* = 0.1979; IFN-γ: *P* = 0.0061; MCP-1: *P* = 0.0474; TNF-α: *P* = 0.2654; Fig. 8b) in INI-0602-treated mice compared with control-group mice. Serum cytokine/chemokine levels did not show any significant differences between the two groups (Supplementary Fig. S6b).

### In vitro INI-0602 administration suppresses inflammatory cytokines/chemokines produced by primary astroglia treated with activated microglia-derived cytokines/chemokines

Under in vivo conditions, astroglia and microglia engage in inseparable and informative crosstalk. We therefore investigated the signals secreted by microglia under inflammatory conditions and whether they can induce the activation of pro-inflammatory astroglia. We also examined whether this crosstalk can be interrupted by blocking Cx. To achieve this, we subjected primary cultured microglia to a 24-h treatment with either vehicle or 1 µg/ml of lipopolysaccharide (LPS). Following this treatment, we replaced the culture media with conditioned medium to remove the residual LPS, and incubated cells for an additional 24 h. Subsequently, we used the harvested conditioned medium from the vehicle-treated microglia (MCM) or LPS-activated microglia (LPS-MCM) to treat purified astroglia. After 24 h, we collected astroglial cell lysates for western blot analysis. Astroglia incubated with LPS-MCM exhibited elevated levels of pro-inflammatory marker proteins and reduced levels of anti-inflammatory marker proteins compared with astroglia treated with MCM (C3: *P* = 0.0006; S100β: *P* = 0.0440; S100A10: *P* = 0.0384; Fig. 8c,d).

To confirm the microglia-secreted signals that induce astroglia to become pro-inflammatory, we conducted a multiplex immunoassay of LPS-MCM and control MCM. LPS-MCM showed increased cytokine/chemokine secretion by microglia compared with MCM from vehicle-treated microglia (IL-1β: *P* < 0.0001; IL-6: *P* = 0.0007; TNF-α: *P* < 0.0001; MCP-1: *P* = 0.0378; Fig. 8e). To further investigate the signals released by pro-inflammatory astroglia into the media, we cultured purified astroglia for 24 h in MCM or LPS-MCM, changed the media to remove each MCM, and continued the incubation for an additional 24 h before collecting culture media. Multiplex immunoassay analysis of the collected media from astroglia after being pre-treated with LPS-MCM and undergoing a media change showed increased levels of IL-1β, IL-6, TNF-α, and MCP-1 compared with MCM pre-treatment (IL-1β: *P* = 0.0028; IL-6: *P* < 0.0001; TNF-α: *P* = 0.0124; MCP-1: *P* = 0.0039; Fig. 8f).

Next, we investigated the role of Cx43 HCs to elucidate the pathway responsible for the release of inflammatory signals from astroglia following microglial activation. We simultaneously treated astroglia with INI-0602 and LPS-MCM and collected cell lysates and culture media after 24 h. Western blot analysis of astroglial cell lysates treated with INI-0602 showed reduced levels of pro-inflammatory marker protein expression and increased levels of anti-inflammatory marker protein expression (C3: *P* = 0.0010; S100β: *P* = 0.0165; S100A10: *P* = 0.0382; Fig. 8c,d) compared with vehicle-treated astroglial cells. Furthermore, multiplex immunoassay analysis of media from astroglia treated with INI-0602 revealed significantly reduced levels of IL-1β, IL-6, TNF-α, and MCP-1 (IL-1β: *P* = 0.04; IL-6: *P* = 0.0188; TNF-α: *P* = 0.0399; MCP-1: *P* = 0.0402; Fig. 8f) compared with cells treated with LPS-MCM only.

These observations demonstrate that activated microglia promote astroglia to release pro-inflammatory signals through activated astroglial HCs, and that this process is significantly diminished by blocking Cx HCs.

### INI-0602 treatment blocks the intracellular uptake and efflux of dye through HCs

**Figure 9:**
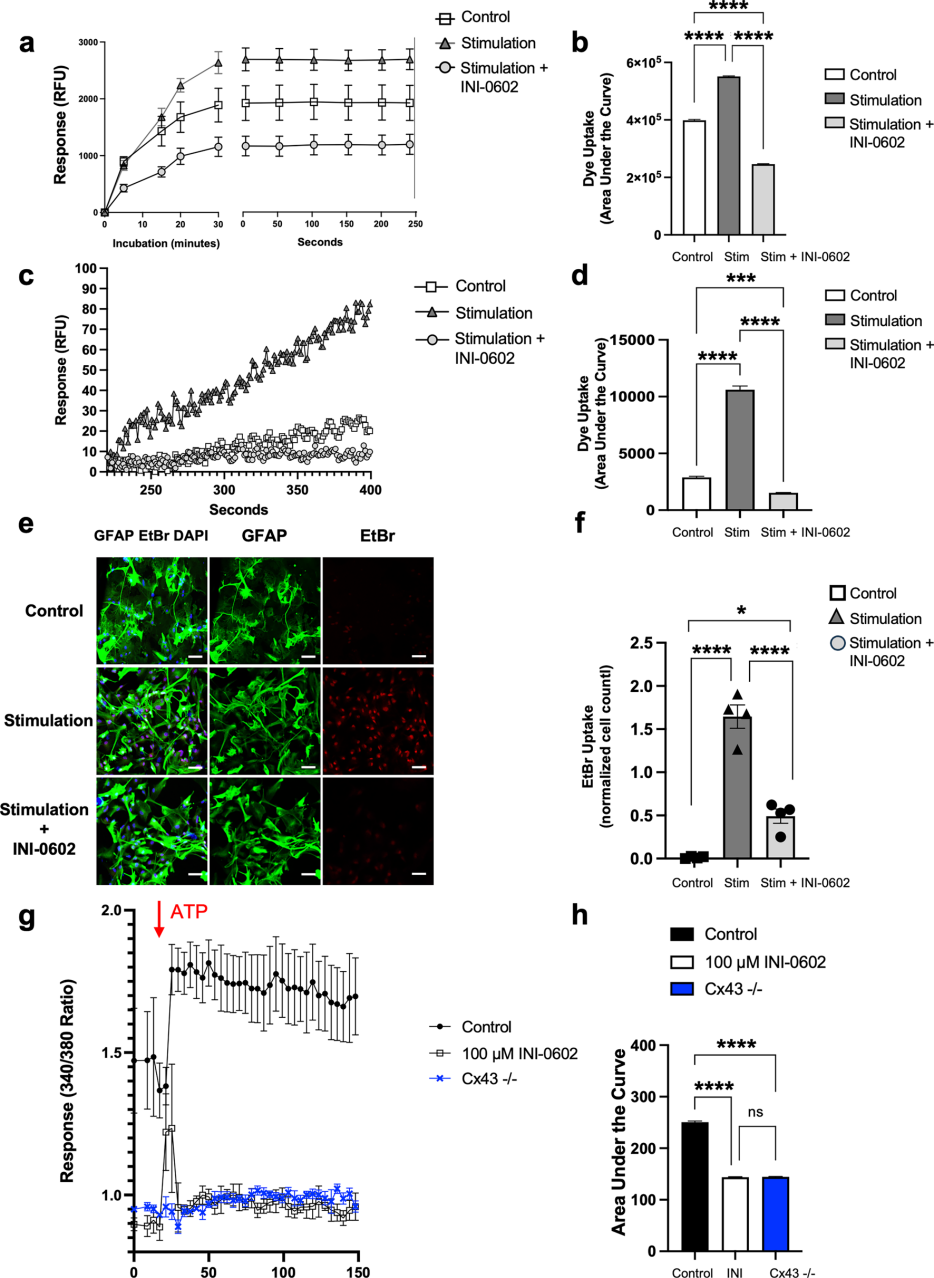
Astroglial HC-mediated dye uptake/efflux responses and calcium propagation are suppressed by INI-0602 treatment. (**a**) Representative data illustrating calcein uptake in response to stimulation, with and without INI-0602 treatment. The kinetics of calcein uptake by astroglia over a 30-min incubation period (left) and an additional 250 s of monitoring past the point of plateau in dye uptake levels (right) are shown. (**b**) Summarized AUC data from normalized calcein uptake in astroglial cells (*n* = 4 separate experiments). INI-0602 was incubated prior to the calcein dye. Statistical data are presented as means ± SEM (n = 4). *P*-values were computed using one-way ANOVA with Bonferroni post hoc tests. ****P < 0.0001. (**c**) Calcein efflux responses in astroglial cells in response to stimulation, with or without INI-0602 treatment. (**d**) Efflux data were computed using the AUC on the graph of the dye uptake curve (0–400 s) (*n* = 4 separate experiments). Statistical data are presented as means ± SEM. *P*-values were computed using one-way ANOVA with Bonferroni post hoc tests. ****P < 0.0001. (**e**, **f**) Immunocytochemical images of primary-cultured astroglia with different treatments [GFAP (green), EtBr (red), DAPI (blue); stimulation: Il-1α (3 ng/ml), TNF-α (30 ng/ml), and C1q (400 ng/ml)] (scale bars: 100 µm). (**f**) Quantification of EtBr-labeled astroglial cell areas from three different groups (*n* = 4 separate experiments). Statistical data are presented as means ± SEM (*n* = 4). *P*-values were computed using one-way ANOVA with Bonferroni post hoc tests. ****P < 0.0001. (**g**) Calcium wave propagation was assessed in Fura-2 AM-loaded astroglial cultures; Fura-2 ratio values were recorded for 150 s. The red arrow indicates the time of ATP injection into the wells. (**h**) Calcium responses in cells were computed using the AUC on the graph (*n* = 4 separate experiments). Statistical data are presented as means ± SEM. *P*-values were computed using one-way ANOVA with Bonferroni post hoc tests. ****P < 0.0001; ns: non-significant.

To determine the effects of INI-0602 treatment on HC activation, we assessed the time course of calcein acetoxymethyl ester (AM) uptake in astroglial cells treated with SM, SM ± in vitro INI-0602, or without any treatment (for the control group). Stimulated astroglia showed significantly elevated levels of dye uptake compared with control cells (*P* < 0.0001; Fig. 9a,b). By contrast, in cells receiving INI-0602 treatment, dye uptake was significantly impaired compared with stimulated cells (*P* < 0.0001; Fig. 9a,b).

Next, we assayed the calcein efflux from astroglial cell cultures preloaded with the AM moiety of this tracer. Accelerated efflux rates were observed in stimulated astroglial cells compared with those of control cells (*P* < 0.0001; Fig. 9c,d) and INI-0602 blocked the calcein efflux from astroglia (*P* < 0.0001; Fig. 9c,d). Moreover, we evaluated astroglial HC activation by ethidium bromide (EtBr) uptake. Stimulated astroglia demonstrated HC activity—as demonstrated by enhanced EtBr uptake compared with that of the control group (*P* < 0.0001; Fig. 9e,f)—that was blocked by INI-0602 treatment (*P* < 0.0001; Fig. 9e,f). These results suggest that INI-0602 influences the influx/efflux of dyes through astroglial HCs, which indicates its modulatory effects under inflammatory conditions.

### INI-0602 acts by blocking astroglial calcium oscillation cascades

We investigated astroglial intracellular calcium ([Ca^2+^]i) changes after treating Cx43^+/+^ and Cx43^−/−^ astroglial cell cultures with adenosine triphosphate (ATP). We employed ATP because astroglia react to ATP with a propagating wave of [Ca^2+^]i increases^22,23^. Experimental analysis revealed that ATP (10 µM) triggered a [Ca^2+^]i spike and subsequent [Ca^2+^]i plateau in Cx43^+/+^ astroglial cells. This response was inhibited by 100 µM INI-0602 treatment [AUC of control (Cx43^+/+^) vs. 100 µM INI-0602 (Cx43^+/+^): *P* < 0.0001; Fig. 9g,h]. Interestingly, calcium propagations of Cx43^−/−^ astroglia were downregulated compared with those of control cells [AUC of control (Cx43^+/+^) vs. Cx43^−/−^: *P* < 0.0001; Fig. 9g,h], and these astroglia showed similar ATP-evoked calcium response values to those observed under INI-0602 treatment [100 µM INI-0602 (Cx43^+/+^) vs. Cx43^−/−^: *P* = 0.9939; Fig. 9g,h], suggesting that the [Ca^2+^]i response, triggered by ATP, is linked to the activation of Cx43 HCs and can be prevented by Cx blockade.

### Astroglia-specific Cx43 ablation in gray matter of the brain reduces Th17 cells but promotes Treg cell migration into the CNS in the peak phase of EAE

**Figure 10:**
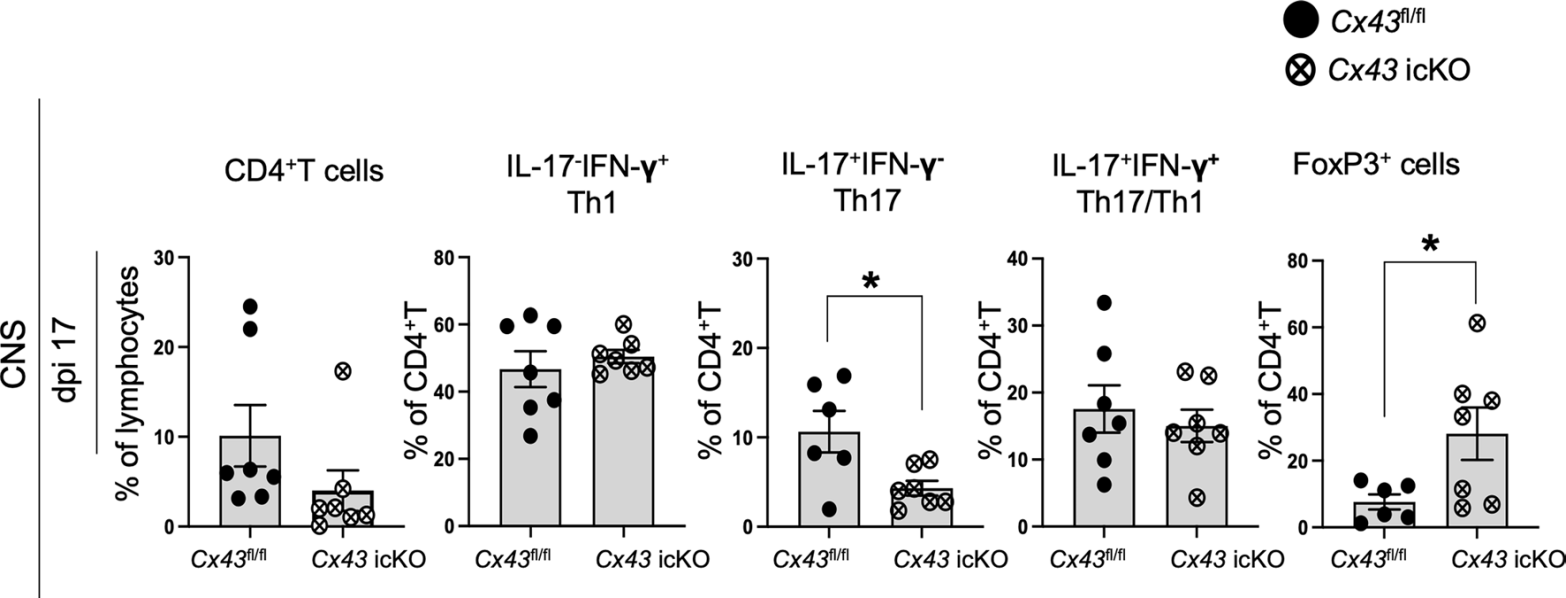
Flow cytometry analysis of T cells isolated from CNS tissues of peak (dpi 17) *GLAST*^+^ *Cx43* icKO or *fl/fl* EAE mice for intracellular cytokine assays and the quantification of individual cell populations. Quantitative analysis of IL-17^−^IFN-γ^+^CD4^+^ (Th1), IL-17^+^IFN-γ^−^CD4^+^ (Th17), IL-17^+^IFN-γ^+^CD4^+^ (Th17/Th1), and FoxP3^+^ cell percentages in CD4^+^ T cells from icKO or *fl/fl* EAE mice at dpi 17. Statistical data are presented as means ± SEM (*n* = 6–7 mice per group). *P*-values were computed using unpaired *t*-tests. *P < 0.05; **P < 0.01.

To elucidate the effect of astroglia-specific *Cx43* ablation on T cell migration in the CNS, we induced EAE in *Cx43*^*fl/fl*^ and *Cx43* icKO mice and measured IFN-γ-producing Th1 cells, IL-17A-producing Th17 cells, and FoxP3^+^ Treg cells by flow cytometry of CD4^+^ T cells (Supplementary Fig. S7a–h displays representative flow cytometry plots illustrating the gating scheme) obtained from CNS tissues of peak (dpi 17) EAE mice from both genotypes for comparison. *Cx43* ablation significantly decreased the IL-17A^+^IFN-γ^−^ Th17 cell percentages in CD4^+^ T cells among the CNS-infiltrating T cells obtained from *Cx43* icKO mice (*P* = 0.1633 for CD4^+^ T cells; *P* = 0.5298 for IL-17A^−^IFN-γ^+^ Th1; *P* = 0.0199 for IL-17A^+^IFN-γ^−^ Th17; *P* = 0.5696 for IL-17A^+^IFN-γ^+^ Th17/Th1; Fig. 10). By contrast, *Cx43* ablation resulted in upregulated FoxP3^+^ Treg cell percentages among the isolated CNS-infiltrating T cells from *Cx43* icKO mice (*P* = 0.0399; Fig. 10).

## Discussion

This research presents four key findings: first, the suppression of astroglial Cx43 overexpression by INI-0602 treatment in chronic EAE. Second, the significant reductions in demyelination, microglial activation, and immune cell infiltration in the spinal cord of EAE mice following INI-0602 treatment. Third, in vitro evidence of the direct anti-inflammatory impact of INI-0602 on astroglia, and fourth, the downregulation of calcium wave propagation in astroglia through the INI-0602-mediated inhibition of HC activation.

Previous studies have reported altered Cx43 parameters in response to various CNS injuries^4,24^. A prior report from our laboratory demonstrated increased expression of Cx43 and the absence of its counterpart, Cx47, in chronic MS lesions^8^. Mirroring the findings from MS patient samples, we observed changes in Cx43 and Cx47 distribution in the lumbar spinal cord throughout the EAE course. EAE induction reduced oligodendroglial Cx47 expression in chronic EAE, whereas astroglial Cx43 was overexpressed. As a result of this imbalance in Cx positioning, the formation of HCs from overexpressed Cxs disrupts glial syncytium. Moreover, pro-inflammatory triggers secreted from HCs on the surface of reactive astroglia accelerate inflammation and promote demyelination^25,26^.

In MS, demyelination plaques are encircled by reactive astroglia. Extensive focal reactive astrogliosis occurs in white matter and, in some areas, in gray matter^27^. Demyelinating lesions also show reduced intensity of synaptic vesicle protein markers, such as synaptophysin and synapsin I, in the gray matter of the spinal cord, alongside the loss of anterior horn neurons^28^. This reduction in synaptophysin immunoreactivity has also been observed in gray matter during acute EAE, with partial recovery during post-EAE^29^. Astroglial Cx43 is involved in sustaining synaptic transmission under physiological conditions by mediating the control of glutamate reuptake and ion homeostasis. However, during inflammatory conditions such as EAE, astroglia contribute to synaptic degeneration by promoting excitotoxic synaptopathy as the result of reduced glutamate uptake by inflammation-activated astroglia^30^. In amyotrophic lateral sclerosis, motor neuron excitotoxic synaptopathy in the gray matter is associated with neuronal Cx36 expression^31^. Given the important role of astroglia and Cxs in shaping synaptic transmission in the gray matter of the spinal cord, we therefore anticipated a substantial influence of inflammation-activated astroglial HCs on synapse formation between neurons in both EAE and MS. In the present study, synaptic vesicles were recovered with INI-0602 treatment; however, the precise relationship between astroglial Cx43 and synaptic hemostasis remains unclear.

In EAE establishment, peripheral leukocytes crossing the BBB are considered the primary cause of demyelination^32,33^. The detection of reactive astrogliosis at this early stage indicates that reactive astroglia play a role in disrupting BBB maintenance and recruiting peripheral immune cells to the CNS^34,35^. Recent studies have highlighted the involvement of Cx43 in BBB endothelial cells and astroglia; their calcium dynamics, mediated by the opening of HCs, are important for triggering barrier leakage during systemic inflammation^36^. These results are further supported by the inhibitor effect of in vivo and in vitro Cx blockade on bradykinin-triggered BBB leakage, which was shown by inhibiting Cx HCs in both endothelial cells and astroglia^37^. In the present study, INI-0602 limited demyelination during the acute phase of EAE by reducing immune factor secretion, thereby influencing the recruitment of Th17 cells into the CNS and potentially contributing to the restoration of BBB integrity, while restricting its disruption by reactive astroglia. Nonetheless, further research is required to explore the relationship between tight junctions and astroglial GJs in BBB maintenance.

We hypothesize that the shift toward a pro-inflammatory environment may have led to an elevation in the activation of HCs on astroglia, and may have also promoted the formation of new HCs from pre-existing Cx43 on the cell surface. To validate this hypothesis, we used a calcein fluorescent probe, which is commonly used to investigate HC function in various cell types (including astroglia) because of its permeability through Cx HCs^38^. We observed calcein efflux to assess HC activity during the shift to an inflammatory environment with stimulation and INI-0602 treatment. Our hypothesis was supported by the in vitro results, which indicated increased calcein dye uptake and efflux, even though the levels of existing Cx43 protein remained unchanged following pro-inflammatory stimulation compared with control cells. Additionally, INI-0602 treatment downregulated Cx43 protein levels, thereby preventing the formation of new HCs, even under pro-inflammatory conditions. Consequently, this led to downregulated calcein efflux from INI-0602-treated stimulated astroglia compared with untreated stimulated astroglia.

Given that we confirmed the inhibitory effect of INI-0602 on Cx43 protein expression levels, we also examined mRNA *Cx43* levels under varying inflammatory conditions. Notably, Cx43 protein levels exhibited only a slight decline in response to C1q, IL-1α, and TNF-α treatment for 24 h, in contrast to the prompt reduction observed in *Cx43* mRNA. A previous study using primary astroglial culture revealed a similarly delayed decline in Cx43 protein levels with LPS treatment (from 18 to 72 h) in contrast to the prompt reduction observed in *Cx43* mRNA (which commenced after 3 h of LPS treatment)^39^. Together, these results suggest that the subacute response of Cx43 protein levels to inflammation tends toward decreased levels, whereas the *Cx43* mRNA response is more robust and rapid. Furthermore, treating cells with INI-0602 following C1q, IL-1α, and TNF-α treatment did not alter the Cx43 mRNA levels, suggesting that INI-0602 does not affect Cx43 transcription levels.

In chronic EAE and MS, lymphocyte infiltration decreases, and glia become the main source of axonal degeneration by inducing the release of reactive oxygen species and inflammatory cytokines^40^. Reactive astrogliosis has a persistent role in disease progression through various mechanisms; it inhibits remyelination and axonal regeneration by forming glial scars and increasing sensitivity to ATP and elevated calcium oscillations^40,41^. In a prior study, excess extracellular cholesterol in the environment—which can be attributed to demyelination—led to elevated calcium oscillations in astroglia that were partially mediated by HC activity; however, it did not have this effect in microglia or neurons^42^. Another study has emphasized the importance of elevated calcium levels at EAE onset, reporting that Th17 cells are able to migrate within the leptomeninges while consistently exhibiting calcium signaling^43^.

Taking this evidence into account, it may be that during demyelination and inflammatory cell infiltration in EAE or MS, astroglia perceive inflammatory alterations in the environment—specifically shifts and elevations in calcium levels—and increased external calcium levels then enhance astroglial Cx43 HC activity^44^. This may then trigger adjacent astroglia activation, setting off a “domino effect” of calcium signaling that significantly impacts neuronal and synaptic activity regulation. These oscillations during EAE create a detrimental cycle of neurotoxic astroglial activation, causing inflammatory damage^45^.

ATP receptors trigger transient increases in [Ca^2+]^i in astroglia via Cx HCs and purinergic signaling (primarily via P2Y receptors)^46–48^; however, pannexin channels (also present in astroglia) do not participate in this transient increase^49^. In the current study, we induced a transient increase in [Ca^2+^]i by applying ATP to Cx43-expressing cultured astroglia. By contrast, when the same ATP concentration was applied to cultured astroglia lacking Cx43, the transient increase in [Ca^2+^]i had a lower amplitude. In astroglia lacking Cx43 HC expression, calcium propagations are suppressed compared with Cx43-expressing cells, but are not entirely prevented because other pathways (such as purinergic signaling) maintain calcium waves. Together, these experimental findings highlight the crucial role of Cx43 HC activation in increasing intercellular calcium communication.

In vitro INI-0602 treatment of Cx43-expressing astroglia resulted in a lower amplitude of transient [Ca^2+^]i increase after ATP stimulation compared with control cells. Although INI-0602-treated cells showed a moderately higher increase with ATP application compared with Cx43-lacking cells, there was no significant difference in the calcium propagation levels, recorded for 150 s. This difference in response might arise because, unlike Cx43-lacking cells (which are devoid of Cx43 HCs), INI-0602 treatment of Cx43-expressing cells does not completely inhibit all channels, thus potentially retaining some astroglial HC expression.

Our findings suggest that INI-0602 treatment may effectively regulate excess calcium oscillations and astroglia HCs during inflammation, potentially reducing the ability of astroglia to promote inflammation. Enhanced GJ/HC-mediated calcium propagation in reactive astroglia is closely related to multiple aspects of neurological disease^49–52^. Thus, the blockade of this cascade by INI-0602 may contribute to the enhancement of neuroprotective effects.

Demyelination in the CNS is associated with both infiltrating T cell-driven and astroglia-regulated inflammatory responses^53^. Reactive astroglia release pro-inflammatory cytokines/chemokines—including IL-6, IFN-γ, TNF^5,54,55^, and MCP-1^53,55^—that can upregulate recruitment of T cells to the CNS^5,54,57^ and prompt T cells to adopt a detrimental phenotype during autoimmunity^58,59^. In response to these findings, we investigated these inflammatory biomarkers in the CSF of EAE mice. Our analysis revealed significant suppression of IL-6 in INI-0602-treated mice on dpi 24. This IL-6 suppression is correlated with downregulated Th17 percentages in INI-0602-treated mice on dpi 24 because IL-6 is known to potentiate inflammation by promoting Th17 cell differentiation^60^. Analysis from dpi 50 revealed significant suppression of IFN-γ and MCP-1 levels in INI-0602-treated mice. CNS-infiltrating CD4^+^ T cells are the major sources of IFN-γ, which is essential not in the induction but in the evolution of the autoimmune attack^61,62^. During CNS inflammation, IFN-γ is the primary regulator of MCP-1, a typical T cell and macrophage chemoattractant^63^. These findings suggest that the suppression of IFN-γ and MCP-1 levels in the CSF is consistent with the suppression of IFN-γ-secreting T cells by INI-0602 on dpi 50.

LPS indirectly opens astroglial HC by activating microglia, which release TNF-α and IL-1β^44,64^. Similarly, our results showed TNF-α, IL-1β, IL-6, and MCP-1 secretion from LPS-induced microglia and from astroglia treated with LPS-induced MCM; notably, simultaneous INI-0602 treatment of astroglia suppressed these cytokine/chemokine secretion levels.

Pannexin 1, an ATP-releasing channel expressed on vasculature, astroglia, and neurons, opens in response to endothelial cell activation by inflammatory mediators such as TNF-α^65^. It plays a crucial role in intercellular communication within the vasculature and among astroglia. In the present study, we did not inhibit pannexins; however, their activation leading to ATP release might be involved in Cx-mediated pro-inflammatory release during inflammation.

Taken together, INI-0602 treatment might prevent pro-inflammatory crosstalk between microglia and astroglia and cytokine/chemokine secretion.

A previous study showed that transgenic inhibition of astroglial reactivity results in the decreased production of TNF, IFN-γ, and IL-17 by CD4^+^ T cells in the acute phase of EAE^53^. We examined the modification of T cell infiltration into the spinal cord during the peak of EAE in the absence of gray matter astroglia-specific Cx43 using *GLAST*^+^ *Cx43* icKO mice. These mice showed reduced IL-17^+^IFN-γ^−^ Th17 cell percentages in CD4^+^ T cells as well as elevated FoxP3^+^ Treg cells compared with *GLAST*^+^ *Cx43*^*fl/fl*^ mice. Th17 cells play a pivotal role in establishing EAE, whereas Treg cells limit Th17 cell function by inhibiting calcium signaling and blocking access to antigen-presenting cells in the spinal cord, thereby preventing their reactivation^43^. The alleviation of EAE signs and decreased immune cell infiltration in the CNS of *GLAST*^+^ *Cx43* icKO mice may be attributed to the inhibition of astroglial HC activation. This inhibition resulted in higher Treg profiles and reduced accumulation of Th17 profiles in the CNS. It may thus be beneficial to further investigate the resident glial responses in *GLAST*^+^ *Cx43* icKO EAE mice.

TPPP is exclusively found in mature oligodendroglia, and is responsible for forming a dense myelin sheath around axons^66^. Upregulated TPPP expression has been detected in MS patients with remyelinating lesions^67^. In our study, EAE mice displayed increased TPPP^+^ oligodendroglia compared with WT mice, potentially suggesting an inflammatory response to axonal damage in EAE lesions. Additionally, EAE mice treated with INI-0602 exhibited significantly higher TPPP expression during the acute phase compared with mice receiving vehicle treatment. We therefore speculate that INI-0602 treatment might aid in remyelination by suppressing inflammatory glial activation and regulating TPPP expression during oligodendroglial maturation.

## Conclusion

The blockade of Cx43 HC activity in chronic lesions of MS patients might exert a therapeutic effect by suppressing neuroinflammation exacerbated by upregulated astroglial Cx43, and by inhibiting excessive immune cell infiltration into the CNS.

## Methods

### Ethical statement

The experimental protocols were formulated with careful consideration to reduce animal use in research and alleviate animal distress and suffering. Each experiment was handled in compliance with the guidelines published by the Science Council of Japan, in addition to the animal research guidelines set by ARRIVE (Animal Research: Reporting of In Vivo Experiments). The Animal Care and Use Committee of Kyushu University provided ethical approval for the study (#No. A23-059–0). We confirm that all experiments were performed in accordance with relevant guidelines and regulations.

### Animals and genotyping

C57BL/6 (B6) female mice were acquired from Kyudo Co. (Tosu, Japan) and given a week to acclimatize to the animal facility environment prior to immunization; they had ad libitum access to food and water.

Dr. M. Götz from the University of Ludwig-Maximilians provided us with *GLASTCreER*^*T2*^ mice on a B6 background^68^. *Cx43* conditional “floxed” mice (*Cx43*^*fl/fl*^ mice) on a B6 background were acquired from Jackson Laboratory (Bar Harbor, ME, USA). We bred *GLASTCreER*^*T2*^ mice with *Cx43*^*fl/fl*^ mice to produce *GLASTCreER*^*T2*^;*Cx43*^*fl/fl*^ mice. We performed genotyping by amplifying DNA purified from fresh mouse tissue (ear punch) using PCR with the following primers: *GLAST* F8 (5′-GAG GCA CTT GGC TAG GCT CTG AGG A-3′), *GLAST* R3 (5′-GAG GAG ATC CTG ACC GAT CAG TTG G-3′), and *CreERT2* (5′-GGT GTA CGG TCA GTA AAT TGG ACA T-3′). The amplicons of the *GLASTCreER*^*T2*^ and WT alleles were 410 bps and 720 bps, respectively.

### Knockdown of *Cx43* in GLAST^+^ astroglia

To knock down astroglial *Cx43* in gray matter, *GLASTCreER*^*T2*^;*Cx43*^*fl/fl*^ (*Cx43* icKO) mice received IP injections of 1 mg of tamoxifen (Sigma-Aldrich, St. Louis, MO, USA) in 100 µl of corn oil twice daily for 5 consecutive days. An identical procedure and dosage were employed to administer tamoxifen to the control mice (*Cx43*^*fl/fl*^).

### Induction and monitoring of EAE

Eight-week-old female B6 WT mice (weighing approximately 20 g) were injected with MOG_35–55_ peptide, as described previously^69^. Briefly, on day 0, subcutaneous injections were administered to mice to deliver 200 µg MOG_35-55_ peptide in 50 µl of 20 nM glycine–HCl (pH 3.0) mixed with an equal quantity of Complete Freund Adjuvant containing 10 mg/ml *Mycobacterium tuberculosis* H37RA (7027; Chondrex, Woodinville, WA, USA), and an IP injection delivering 200 ng pertussis toxin (168-22471; Wako, Osaka, Japan) in 0.2 ml of phosphate-buffered saline (PBS). A subsequent IP pertussis toxin (200 ng/mouse) injection was administered 48 h after the initial injection. For disease monitoring, the mice were weighed and scored daily. The scale of scoring was as follows: 0, normal; 1, tail paralysis; 2, tail paralysis and hind limb weakness; 3, complete hind limb paralysis; 4, hind limb and forelimb paralysis; 5, moribund.

### INI-0602 treatment

For in vivo experiments, INI-0602 (INI, Nagoya, Japan) was suspended thoroughly in 5% volume of 100% EtOH and 95% volume of saline using an agate mortar as previously described^15^. INI-0602 (40 mg/kg) was administered to B6 mice via IP injection on alternate days, beginning on dpi 17 and continuing until dpi 24 for acute phase experiments or from dpi 17 to 50 for chronic phase experiments. Equivalent volumes of saline solution were given to the control group (vehicle treated). For in vitro experiments, INI-0602 was dissolved in dimethyl sulfoxide (final concentration: 0.25%) as previously described^15^.

### Tissue preparation

Mice were anesthetized with 3% isoflurane (Pfizer Japan Inc., Tokyo, Japan) before being euthanized by exsanguination via transcardial perfusion with PBS, followed by ice-cold 4% paraformaldehyde (PFA) in 0.1 M PBS. The spinal cord was collected at dpi 24 and 50, fixed overnight in 4% PFA at 4 °C, and processed for paraffin sectioning at 4-µm thickness or cryostat sectioning at 20-µm thickness.

#### 3,3′-Diaminobenzidine (DAB) staining and imaging

Immunohistochemistry was performed on paraffin-embedded lumbar spinal cord sections using an indirect immunoperoxidase technique. Following deparaffinization, endogenous peroxidase was quenched using 0.3% hydrogen peroxide in absolute methanol for 30 min. Sections were then permeabilized with PBS containing 0.1% Triton for 10 min, washed with Tris–HCl for 5 min, soaked in 10 mM citrate buffer, and autoclaved at 120 °C for 10 min. Once cooled to room temperature, sections were incubated with primary antibodies overnight at 4 °C. The next day, rinsed sections were subjected to enhanced indirect immunoperoxidase labeling using an Envision kit (K4001, K4003; Dako, Glostrup, Denmark), and to color reaction using DAB tetrahydrochloride (MK210; Takara, Tokyo, Japan). After being counterstained with hematoxylin, the stained sections were photographed using a light microscope (Olympus, Tokyo, Japan).

#### Immunofluorescent staining and imaging

Sections were deparaffinized through xylene and ethanol. They were then autoclaved in 10 mM citrate buffer at 120 °C for 10 min. After cooling, the sections were blocked in 5% normal goat serum in PBS with 0.5% Triton X-100 before being incubated with primary antibodies overnight at 4 °C. Details of the primary antibodies are provided in Supplementary Table 1. The next day, the sections were washed before being incubated with Alexa 488‐conjugated goat anti‐mouse IgG and Alexa 546‐conjugated goat anti‐rabbit IgG (Invitrogen, Waltham, MA, USA) secondary antibodies for 2 h. 4′,6-diamidino-2-phenylindole (DAPI) was used for nuclear staining. Fluorescent images were obtained using a confocal laser microscope system (Nikon A1; Nikon, Tokyo, Japan). During image acquisition, consistent microscopic settings were implemented for all images within each experimental set, and images were captured from corresponding regions. We used sequential scanning to minimize the effects of crosstalk, and used line averaging and a slow scan speed.

### Image analyses

To quantify cell infiltration, sections from the L4–5 spinal cord levels were examined. Fiji-ImageJ analyzing tools (National Institutes of Health; acquired from https://imagej.net/ij/index.html) were used with publicly accessible plugins: JACoP BIOP Version and FracLac.

To quantify fluorescent intensity, we determined the areas stained with synaptophysin, GFAP, Iba-1, F4/80, Cx47, and TPPP by converting them to grayscale and applying a uniform threshold across all stained tissue for each antibody. Subsequently, the pixel intensity of protein densities (integrated density value) was calculated proportional to the total area of the imaged frame. For double staining (C3-GFAP, Cx43-GFAP, S100A10-GFAP, and S100β-GFAP), we quantified the overlapping area using the Manders method in ImageJ Fiji software with the JACoP plugin. The area corresponding to the fraction of the green signal that overlapped with the red signal was then divided by the total area to calculate the overlapping area percentage, which was used for statistical analysis.

The DAB and hematoxylin staining were first deconvoluted; the separated images then underwent measurement of mean gray values with the application of a uniform threshold across all stained tissue. The number of nuclei in a field was determined from hematoxylin staining by analyzing particle numbers using ImageJ Fiji software. For CD3 staining, cells that were stained with CD3 and had hematoxylin-stained nuclei were counted; the data were recorded for statistical analysis to compare between groups.

### MBP immunodensity

MBP staining signal was quantified by measuring the mean gray value of a designated area. The number of nuclei within the same area was determined using hematoxylin staining and particle analysis. To normalize DAB staining to the number of nuclei, the mean gray value was divided by the number of nuclei in the quantified area for each image.

### Acquisition of EtBr-labeled cells

For quantification of EtBr-labeled astroglia, cells were stained with GFAP, and those containing EtBR-labeled nuclei were counted. The values were normalized by dividing by the total cell count in the entire sample area.

### Circularity analysis of microglia

Circularity analyses of single microglial cell images were processed automatically using ImageJ software and the FracLac plugin, based on previously described criteria^70^. Briefly, randomly selected microglia were converted into binary images, and a region of interest was chosen to encompass all microglia with branches (Fig. S3a,b). Special attention was paid to excluding branches that did not belong to the selected cell, using the original image as a reference. After isolating a microglial cell, the binary image was transformed into an outline (Fig. S3c). The FracLac plugin was then used for cell analysis, employing 'box counting' and setting the 'grid design Num G′ to 4. Microglial circularity was then measured (Fig. S3d).

### T cell proliferation assay

In each well of a 96-well plate, 100 µl splenocyte emulsion (1 × 10^6^ cells/well) was added, followed by 100 µl Roswell Park Memorial Institute (RPMI) medium including either 2.5, 12.5, or 25 µg/ml MOG_35-55_, or 100 µl of RPMI medium as a blank control. The plate was then incubated for 72 h at 37 °C in a humidified environment containing 5% CO_2_. For the final 18 to 24 h of incubation, 20 µl of bromodeoxyuridine (BrdU) solution was applied to each well (except wells designated as background controls, without BrdU). At the end of the 72-h incubation, an assay was performed using a BrdU kit (ab126556; Abcam, Cambridge, UK) as per the manufacturer’s instructions. Briefly, the cells were fixed for 30 min, incubated at room temperature with 100 µl of anti-BrdU monoclonal detector antibody for 1 h, incubated with 100 µl of peroxidase-conjugated goat anti-mouse IgG for 30 min, and then incubated with 100 µl of tetramethylbenzidine peroxidase in the dark for 30 min, followed by the addition of 100 µl stop reaction solution. The absorbance measurements of the wells were taken at wavelengths of 450/560 nm using a microplate reader (MTP-800AFC; Corona Electric, Hitachinaka, Japan).

### Flow cytometry

EAE mice were injected with or without INI-0602 (40 mg/kg) from dpi 17 to 24 (for the acute phase) and dpi 17 to 50 (for the chronic phase). On dpi 24 or 50, mice were perfused with 30 ml PBS via the cardiac route immediately after euthanasia. Brain and spinal cord tissues were collected and homogenized using a glass homogenizer. A 30%/70% Percoll (GE Healthcare, Chicago, IL, USA) gradient was created to separate mononuclear cells, as previously reported^71^. At the same time, aseptically extracted spleens were disintegrated into splenocytes and individual cells to be suspended in complete RPMI medium [RPMI-1640 medium enriched with 2 mM L-glutamine (#G7513; Sigma-Aldrich), 1 mM sodium pyruvate (Gibco, Thermo Fisher Scientific, Waltham, MA, USA), 50 µM 2-mercaptoethanol, 50 U/ml penicillin, 50 µg/ml streptomycin, and 10% fetal bovine serum (#193,012; Sigma-Aldrich)], as well as the mononuclear cells. To analyze CD4^+^ T cell subsets (Th1, Th17, Th17/Th1, and Treg cells), splenocytes or CNS-infiltrating mononuclear cells (2 × 10^6^ cells/well) were placed in 96-well plates and stimulated using 0.1 mg/ml phorbol 12-myristate 13-acetate, 0.5 mg/ml ionomycin, and 5 mg/ml brefeldin A (Sigma-Aldrich). To define a positive/negative threshold for cytokine response, unstimulated samples were evaluated for each sample. At the end of 4 h of incubation, the cells were placed into 1.5-ml tubes and stained with PE/Cy7-I-A/I-E (clone M5/114.15.2; BioLegend, San Diego, CA, USA), fluorescein isothiocyanate-CD4 (clone RM4-5; BioLegend), APC-IL-17A (clone TC1118H10.1; BioLegend), phycoerythrin-Foxp3 (clone FJK-16s; Invitrogen), and PerCP/Cy5.5-IFN-γ (clone XMG1.2; Invitrogen) after washing. The percentages of IL-17^−^IFN-γ^+^CD4^+^ T cells (reflective of Th1 cells), IL-17^+^IFN-γ^−^CD4^+^ T cells (reflective of Th17 cells), IL-17^+^IFN-γ^+^CD4^+^ T cells (reflective of Th17/Th1 cells), and CD4^+^Foxp3^+^ T cells (reflective of Treg cells) among CD4^+^ T cells were measured using a BD FACSVerse flow cytometer (BD, Franklin Lakes, NJ, USA) and evaluated using FlowJo software (Tree Star, Ashland, OR, USA).

### Primary glial cell cultures

To prepare primary mixed glial cell cultures, the brains of neonatal (postnatal day 2) B6 mice were collected under sterile conditions and positioned in ice-cold Hanks’ balanced salt solution (HBSS; Sigma-Aldrich) mixed with 50 U/ml penicillin and 50 µg/ml streptomycin (Gibco) to eliminate contamination. The following experimental steps, described previously^72^, involved the removal of brain tissue meninges and the separation of the tissue into smaller pieces using nylon mesh. Next, the cell solutions of two brains were placed in a 75 cm^2^ culture flask containing 10 ml glial media and incubated at 37 °C in a humidified environment containing 5% CO_2_. Glial media was a mixture of Dulbecco’s Modified Eagle’s Medium (Sigma-Aldrich) enriched with 10% fetal bovine serum (Equitech-Bio, Kerrville, TX, USA), 5 µg/ml bovine insulin (Sigma–Aldrich), and 0.2% glucose. The medium was replaced during the initial week, and no replacements were made for the subsequent 2 weeks to induce microglial proliferation. On days 12–15, mixed glial cells that reached confluency were disengaged with Accutase treatment (Innovative Cell Technologies, San Diego, CA, USA) and subcultured to reach confluency; they were used in experiments after 5–10 days.

To purify astroglia and microglia from the mixed glial cell culture, we used magnetic-activated cell sorting (MACS) (Miltenyi Biotec, Bergisch Gladbach, Germany) following a previously described method^72^. On days 12–15, mixed glial cells were disengaged using Accutase, rinsed, and suspended in 10 ml MACS Separation Buffer (Miltenyi Biotec). They were then passed through a 70-µm pore filter and centrifuged at 300 × *g* for 5 min at 4 °C. Next, 10 µl CD11b MicroBeads (microbeads coupled to a monoclonal rat anti-mouse CD11b antibody; Miltenyi Biotec) and 90 µl separation buffer were placed into tubes containing 1 × 10^7^ cells and incubated for 30 min at 4 °C. Following washing, the cells were resuspended in 500 µl separation buffer per 1 × 10^8^ cells and positioned within an LS column (Miltenyi Biotec) installed in a QuadroMACS™ cell separator (Miltenyi Biotec) for segregation into CD11b^+^ and CD11b^−^ segments. The CD11b^−^ segment (which is the astroglia-enriched segment) was then resuspended in glial media and plated to reach 100% confluency; the cells were used in experiments after 7–10 days. After separation, astroglia were replaced in 96-well plates at an approximate concentration of 20,000 cells per well for the calcium imaging experiments. The cultures displayed over 95% purity, as confirmed by GFAP, excitatory amino acid transporter (EAAT)1, EAAT2, and Iba-1 immunostaining.

Gray matter astroglia-specific Cx43-deficient (Cx43^−/−^) and WT (Cx43^+/+^) astroglial cultures were generated using brains from neonatal Cx43-deficient or WT mice.

### 4-hydroxy tamoxifen treatment of primary *GLAST*^+^*Cx43* icKO astroglial cell culture

Primary glial cell cultures were derived from the brains of neonatal *GLAST*^+^ *Cx43* icKO mice, or from *GLAST*^+^ *Cx43*^*fl/fl*^ mice for the control group. This was followed by the generation of astroglial cultures using a previously described method^61^. Later, cells were exposed to 2 µM 4-hydroxy tamoxifen (H7904; Sigma-Aldrich) for 72 h.

### Calcium imaging

Astroglia were plated in 96-well plates with black walls and a clear base (Costar, Glendale, Arizona, USA) at a concentration of 20,000 cells per well, and were cultured in medium containing 100 µM INI-0602 for 40–60 min at 37 °C. Untreated or INI-0602-treated astroglia were then incubated with 2 mM Fura-2 AM (ab120873; Abcam) in HBSS for 40 to 60 min at 37 °C. Following this manipulation, buffer was extracted using a multi-pipette, and cells were rinsed for 15 to 20 min using HBSS to wash away extracellular dye. Cells were preheated for 10 min before data collection was initiated. Fura-2 fluorescence was assessed at excitation wavelengths of 340 and 380 nm with an emission wavelength of 520 nm, using a Flexstation 3 plate reader (Molecular Devices, San Jose, CA, USA). A sampling interval of 3 s with 60 reads per well was applied. The Fura-2 ratio was computed using Softmax Pro v5.4 (Molecular Devices), and reactions were quantified by calculating the AUC.

### Immunofluorescent staining of cultured cells

Astroglia were plated at an approximate concentration of 20,000 cells per well in eight-well culture slides (Corning Inc., Corning, NY, USA) and cultured in medium including 100 µM INI-0602 for 40–60 min at 37 °C. After the treatment, the medium was aspirated, and cells were washed with PBS, fixed with 4% PFA for 5 min, and permeabilized with 0.05% Triton X-100 in PBS for 15 min. The cells were then incubated with primary antibodies against Cx43, GFAP, EAAT1, and EAAT2 (comprehensive antibody information is provided in Supplementary Table 1) in 0.05% Triton X-100 in PBS with 5% goat serum for 1 h at 37 °C. Next, the cells were rinsed before being incubated with DAPI and Alexa 488-, 546-, or 594-coupled secondary antibodies for 30 min at 37 °C. Images were acquired using a confocal laser microscope system (Nikon A1) or a conventional fluorescence microscope (BZ-X700, Keyence, Osaka, Japan).

### Collection of CSF and serum

For CSF collection, a glass capillary with a sharpened tip (inner diameter 0.75 mm, outer diameter 1.0 mm) was attached to a syringe. After anesthetizing each mouse, a midline incision was made in the skin over the skull between the ears to expose the cisterna magna under a microscope. The capillary was then precisely inserted into the cisterna magna for CSF retrieval. Collected samples were subsequently stored at − 80 °C.

For serum collection, a 25-G needle with a 1-ml syringe was prepared. After anesthetizing each mouse, a cardiac puncture from the right ventricle was performed to collect serum. Collected samples were placed in a specialized serum separator collection tube for centrifugation and stored at − 80 °C.

### Multiplex fluorescence immunoassay for CSF cytokines, serum samples, and cell media

To assess inflammatory mediators in mouse CSF and serum, we harvested 5 µl of CSF and 30 µl of serum from each mouse. To measure inflammatory cytokine levels in the cell media after all treatments of glial cells, we collected 50 µl of cell media from each sample. The concentrations of eight cytokines (IL-1β, IL-2, IL-6, TNF-α, IFN-γ, IL-17a, IL-10, and MCP-1) were assessed in the CSF and serum samples with a Bio-plex Multiplex System, using a Bio-plex Pro Assay mouse multiplex cytokine kit (Bio-Rad Laboratories, Hercules, CA, USA) as per the manufacturer’s instructions. Samples were duplicated for measurements.

### Glial cell treatment

Mixed glial cell cultures and microglial cultures were subjected to vehicle (0), 1 µg/ml LPS, and 100 µM INI-0602 for 24 h in Dulbecco’s Modified Eagle’s Medium. Astroglial cultures were treated with SM, including IL-1α (3 ng/ml, I3901; Sigma-Aldrich), TNF-α (30 ng/ml, 8902SF; Cell Signaling Technology, Danvers, MA, USA), and C1q (400 ng/ml, 204,876; Sigma-Aldrich) as previously described^73^.

### HC dye uptake assays

Astroglia were loaded with calcein AM (C326, Dojindo Laboratories, Kumamoto, Japan) in HBSS for 40 min at 37 °C. After loading, buffer was extracted using a multichannel pipette and cells were rinsed for 15 to 20 min with HBSS to remove extracellular dye. Following the monitoring of calcein uptake, wells were washed thoroughly to remove extracellular or unbound dye, and the time course of fluorescence emission changes was evaluated relative to the initial fluorescent intensity. Calcein fluorescence was assessed using an excitation wavelength of 485 nm and an emission wavelength of 540 nm with a Flexstation 3 plate reader.

Astroglia were loaded with 0.5 µM EtBr in HBSS for 15 min at 37 °C, washed with warm HBSS, and fixed with 4% PFA for 10 min. Images were obtained within 30 min, and intracellular EtBr fluorescence of single cells was quantified using ImageJ.

### Western blotting

Glial cells were solubilized in buffer, including 0.5% sodium dodecyl sulfate (Nacalai Tesque, Kyoto, Japan) and PhosSTOP phosphatase inhibitor cocktail (Roche Diagnostics, Mannheim, Germany) on ice for 30 min. Next, the lysates were centrifuged at 4 °C for 10 min at 10,000 × *g* and the upper layers were gathered for protein concentration determination using a bicinchoninic acid protein assay kit (Pierce, Thermo Fisher Scientific). The protein concentrations of the samples were adjusted to a uniform level, fused with Laemmli’s buffer, and boiled at 95 °C for 5 min. Subsequently, 10 µg protein per well was segregated using a 4.5%–15% gradient poly-acrylamide gel (Bio-Rad Laboratories) electrophoresis technique and transferred onto polyvinyl difluoride membranes. The membranes were first incubated with a blocking solution and then with the following primary antibodies: anti-C3d (HM1045; R&D Systems, Minneapolis, MN, USA), anti-S100A10 (AF2377; R&D Systems), anti-S100β (ab52642; Abcam), and anti-GFAP (2E1.E9; STEMCELL Technologies, Vancouver, Canada). After washing, the membranes were incubated with horseradish peroxidase-conjugated secondary antibodies for 1 h at room temperature. Following another washing step, the membranes were observed using enhanced chemiluminescence (ECL Prime; GE Healthcare Bio-Sciences AB, Uppsala, Sweden). We quantified band intensities employing the ChemiDoc™ XRS system (Bio-Rad Laboratories) and normalized signals to those of the control (β-actin).

### RNA purification and quantitative real-time RT-PCR

An RNeasy Mini kit (Qiagen, Venlo, Netherlands) was used to isolate total RNA in accordance with the manufacturer’s guidelines. Complementary DNA (cDNA) was produced using ReverTra Ace qPCR RT Master Mix, which includes gDNA Remover (Toyobo, Osaka, Japan). Measurements of cDNA using real-time RT-PCR were conducted using the QuantStudio 3 Real-Time PCR System (Applied Biosystems, Thermo Fisher Scientific) using TaqMan Gene Expression Master Mix and TaqMan Gene Expression Assays [*Cx43* (Gja1), Mm01179639_s1; *Gapdh*, Mm99999915_g1; Applied Biosystems, Thermo Fisher Scientific], using *Gapdh* as an endogenous control gene. The cycling protocol was 50 °C for 2 min, 95 °C for 10 min, and then 40 cycles of 95 °C for 15 s and 60 °C for 1 min. Cycle threshold values were compared with *Gapdh* to evaluate relative expression. The ΔΔCt and fold change (2^−ΔΔCt^) were then calculated to determine alterations in gene expression across different groups.

### Statistical analysis

GraphPad Prism version 9.5.0 (GraphPad Software Inc., San Diego, CA, USA) was used for data analysis. All statistical data are presented as mean values ± standard error of mean values (SEM). We calculated AUCs to assess overall disease severity for each mouse, and *P*-values were analyzed using the non-parametric Mann–Whitney *U* test^11^ (Figs. 1b; S1b; S2a,b). Unpaired *t*-tests were used to compare WT mice with EAE-induced animals or vehicle-treated animals with INI-0602-treated animals (Figs. 1d; 2c,d,f; 3c,f,i; 5; 6e–h,k–m; 8a,b,e; 10; S4r,s; S5r,s; S6a,b). For comparisons involving more than two groups, one-way analysis of variance (ANOVA) was conducted, followed by multiple comparisons using the Bonferroni correction (Figs. 4c,d; 7e,g,h; 8d,f; 9b,d,f,h). Two-way ANOVA was used for Fig. S6c,d. *P-*values < 0.05 were considered significant.

### Ethics approval

All animal experiments were carried out according to the guidelines for the proper conduct of animal experiments published by the Science Council of Japan, and ethical approval for the study was granted by the animal care and use committee of Kyushu University (#No. A23-059-0). Dr. Hideyuki Takeuchi and Dr. Noriko Isobe are Editorial Board Members of Scientific Reports.

### Supplementary Information

Supplementary Information.

## Data Availability

The datasets used and/or analyzed during the current study are available from the corresponding author upon reasonable request. All antibodies used during this study are included in this published article and its supplementary information files.
