# Supplementary material for: Astroglial connexin 43 is a novel therapeutic target for chronic multiple sclerosis model
Source: Sci Rep. 2024 May 13;14:10877. doi: 10.1038/s41598-024-61508-2 (PMC11091090; doi:10.1038/s41598-024-61508-2)
Supplement: Supplementary file 1 — Supplementary Information. [file 41598_2024_61508_MOESM1_ESM.pdf]

Astroglial Connexin 43 Is A Novel Therapeutic Target for Chronic Multiple Sclerosis Model

Ezgi Ozdemir Takase[1], Ryo Yamasaki[1*], Satoshi Nagata[1], Mitsuru Watanabe[1], Katsuhisa Masaki[1], Hiroo Yamaguchi[2], Jun-ichi Kira[3,4], Hideyuki Takeuchi[5,6,7*], Noriko Isobe[1]

[1]*Department of Neurology, Neurological Institute, Graduate School of Medical Sciences, Kyushu University, Fukuoka, Japan*

[2]*School of Physical Therapy, Faculty of Rehabilitation, Reiwa Health Sciences University, Fukuoka, Japan*

[3]*Translational Neuroscience Center, Graduate School of Medicine, and School of Pharmacy at Fukuoka, International University of Health and Welfare, Ookawa, Japan*

[4]*Department of Neurology, Brain and Nerve Center, Fukuoka Central Hospital, Fukuoka, Japan*

[5]*Department of Neurology and Stroke Medicine, Graduate School of Medicine, Yokohama City University, Yokohama, Japan*

[6]*Department of Neurology, Graduate School of Medicine, International University of Health and Welfare, Narita, Japan*

[7]*Center for Intractable Neurological Diseases and Dementia, International University of Health and Welfare Atami Hospital, Atami, Japan*

*Corresponding authors:

Ryo Yamasaki, MD, PhD

Department of Neurology, Neurological Institute, Graduate School of Medical Sciences, Kyushu University, 3-1-1 Maidashi, Higashi-ku, Fukuoka 812-8582, Japan

Phone: +81-92-642-5340

Fax: +81-92-642-5352

E-mail: yamasaki.ryo.510@m.kyushu-u.ac.jp

Hideyuki Takeuchi, MD, PhD

Department of Neurology and Stroke Medicine, Graduate School of Medicine, Yokohama City University, 3-9 Fukuura, Kanazawa-ku, Yokohama 236-0004, Japan

Phone: +81-45-787-2725

Fax: +81-45-788-6041

E-mail: htake@yokohama-cu.ac.jp

SI Figure S1

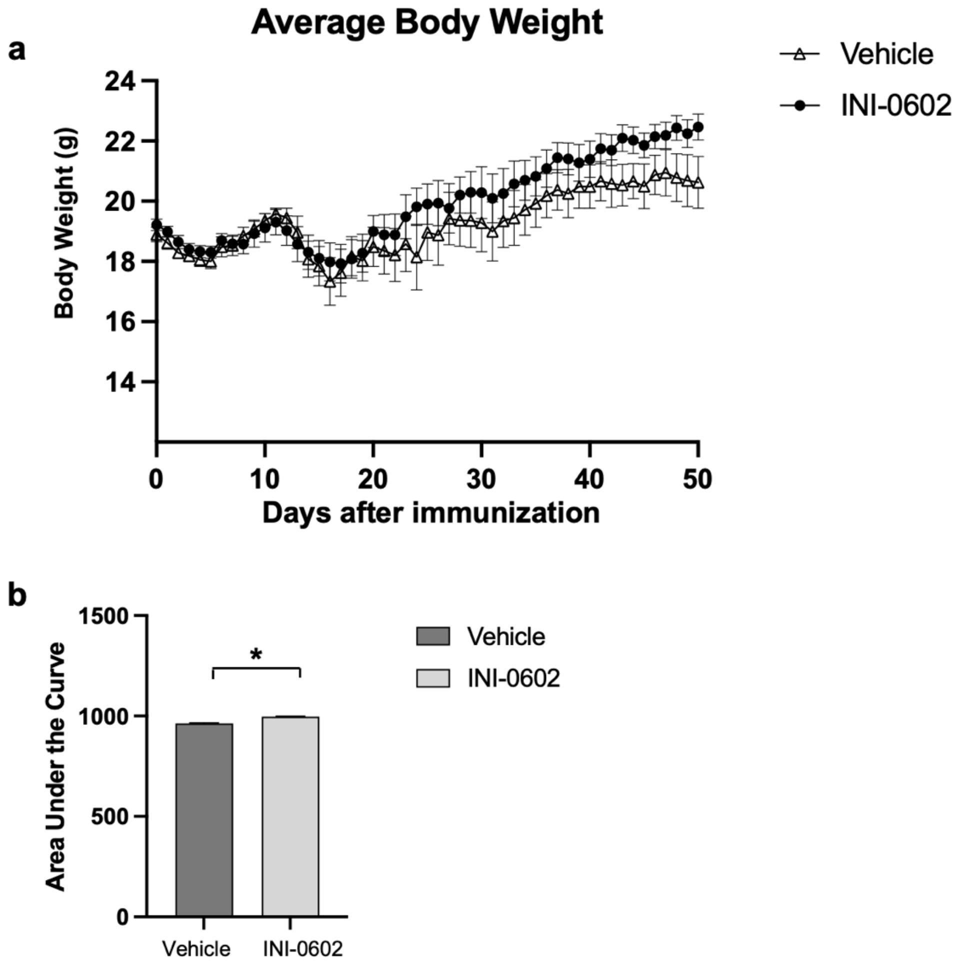
a
Average Body Weight
Vehicle
INI-0602
Body Weight (g)
24
22
20
18
16
14
0
10
20
30
40
50
Days after immunization
b
1500
1000
500
0
Area Under the Curve
*
Vehicle
INI-0602

Supplementary Fig. S1. Body weight loss recovered more quickly in INI-0602-treated mice than in vehicle-treated mice. (a) Body weights (means ± SEM) over time in mice treated with INI-0602 (n = 10) or saline vehicle (n = 10). (b) Body weight change evaluated by comparing the AUC values from dpi 17 to 50. All data are shown as means ± SEM. P-values were calculated using the Mann–Whitney test. *$P < 0.05$.

SI Figure S2

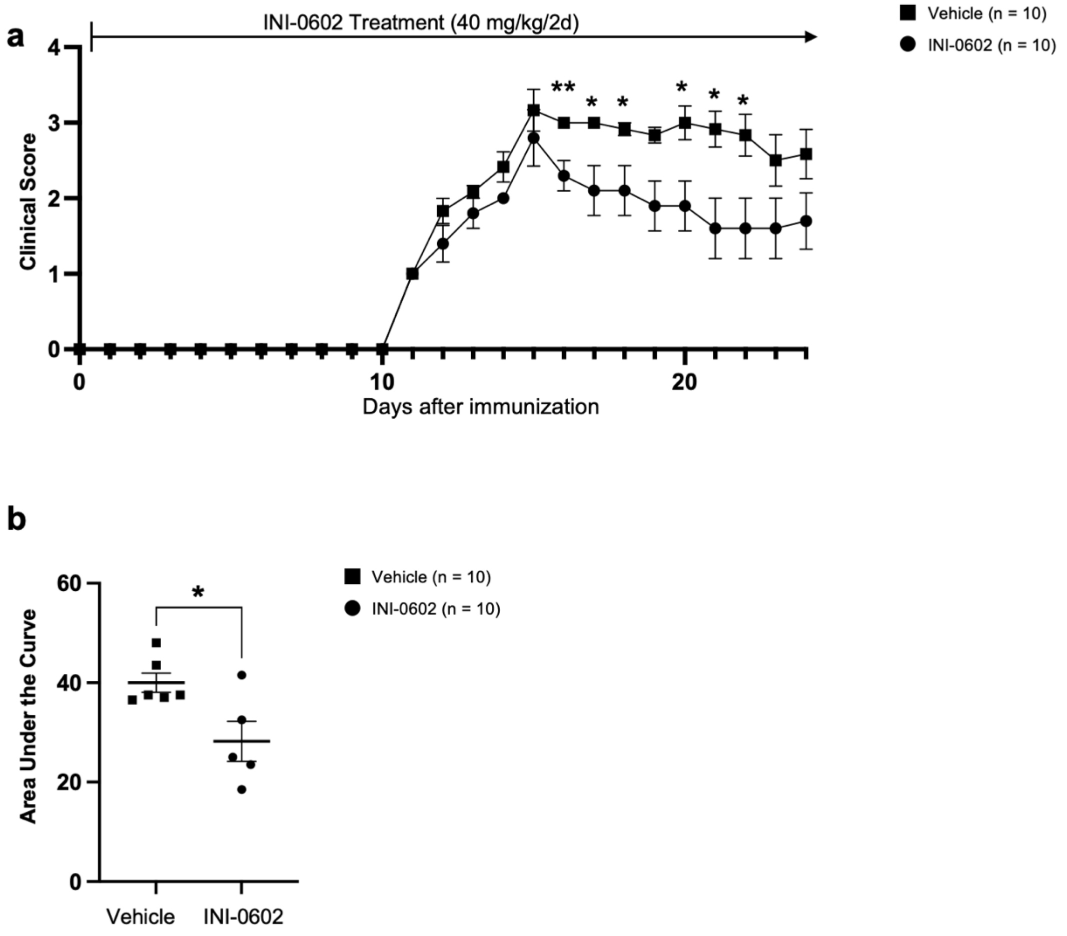
a
INI-0602 Treatment (40 mg/kg/2d)
Vehicle (n = 10)
INI-0602 (n = 10)
Clinical Score
Days after immunization
**
*
0
1
2
3
4
10
20
b
Area Under the Curve
Vehicle
INI-0602
0
20
40
60

Supplementary Fig. S2. (a,b) Clinical scores of EAE mice that received intraperitoneal injection of INI-0602 (40 mg/kg) ($n = 6$) or vehicle control ($n = 5$) from dpi 0 (preventive treatment) (a). AUC values between dpi 0 and 24 (b). Statistical data are presented as means ± SEM. *P*-values were computed using the Mann–Whitney test. $*P < 0.05$.

SI Figure S3

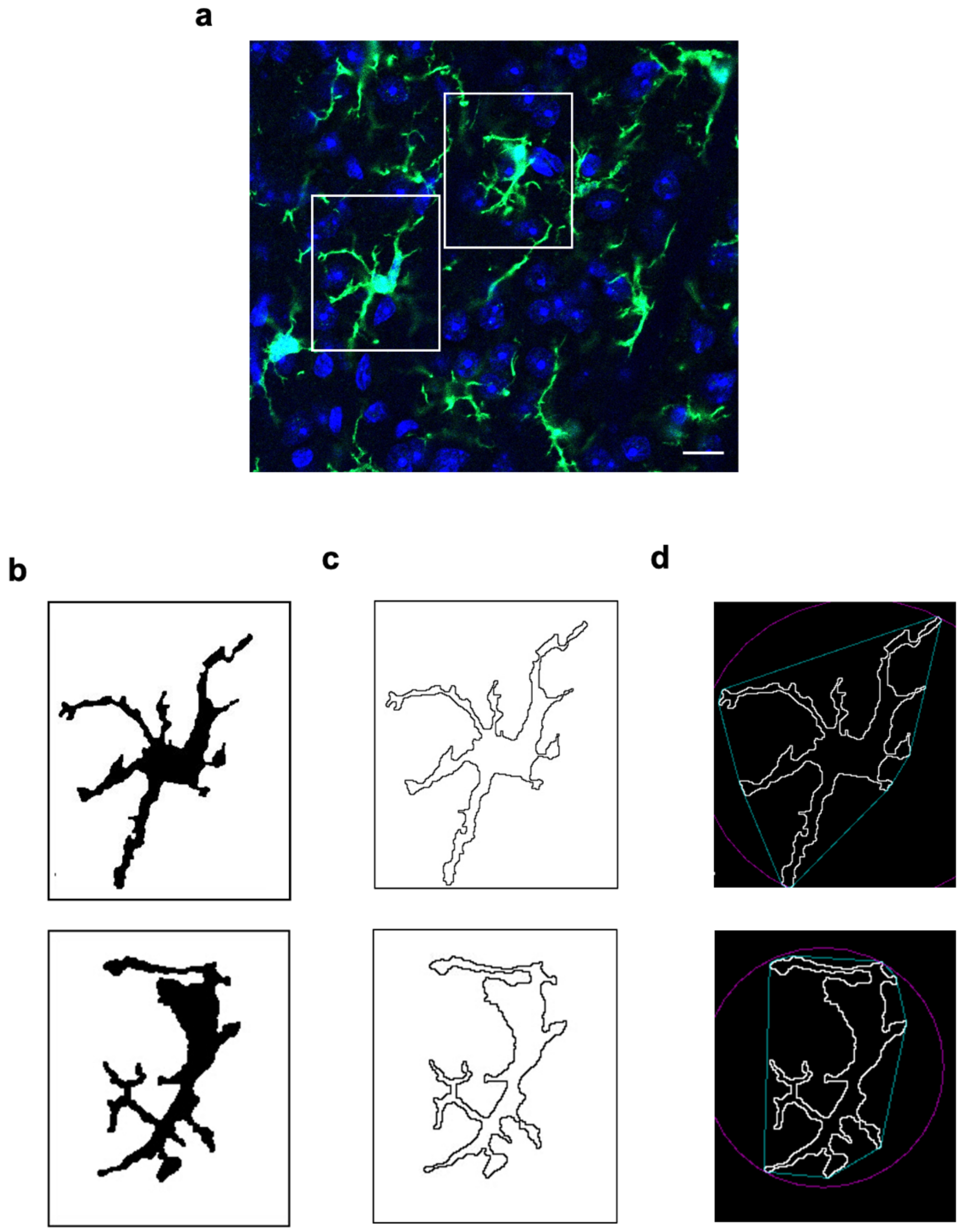

Supplementary Fig. S3. Schematic view of the processing steps for microglial cell circularity assessment. (a) Immunofluorescent image, captured at 40× magnification, displaying Iba-1$^+$ (green) cells in the lumbar spinal cord of EAE mice (scale bars: 10 μm). (b) Randomly selected microglia were converted to binary images with a region of interest chosen to encompass all microglia with branches. (c) The binary image was transformed into an outline. (d) The FracLac plugin was used for cell analysis, employing 'box counting' and setting the 'grid design Num G' to 4 (d).

SI Figure S4

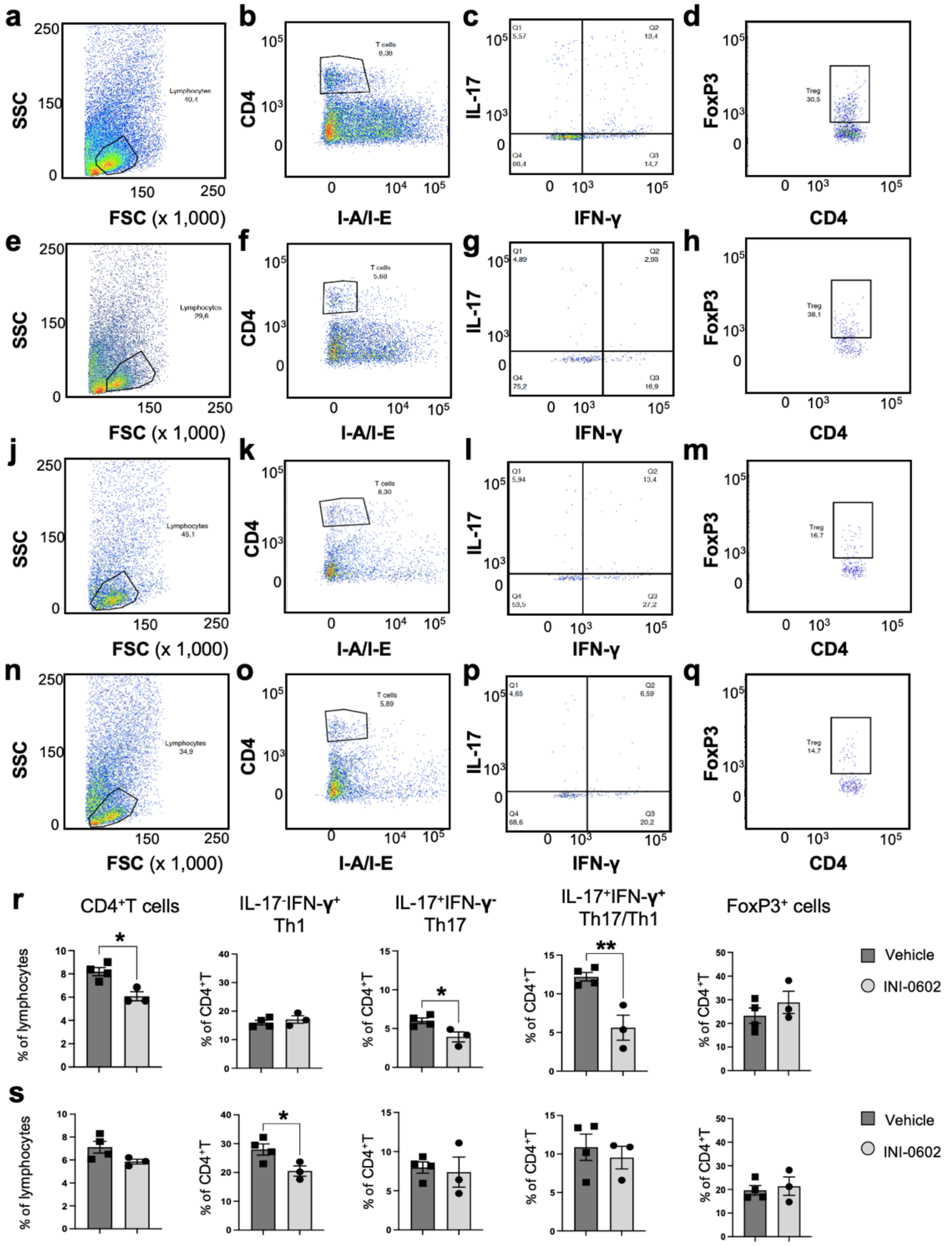
a
b
c
d
e
f
g
h
j
k
l
m
n
o
p
q
r
s
SSC
FSC (x 1,000)
CD4
I-A/I-E
IL-17
IFN-γ
FoxP3
Lymphocytes
T cells
Treg
CD4⁺T cells
IL-17⁻IFN-γ⁺ Th1
IL-17⁺IFN-γ⁻ Th17
IL-17⁺IFN-γ⁺ Th17/Th1
FoxP3⁺ cells
% of lymphocytes
% of CD4⁺T
Vehicle
INI-0602

Supplementary Fig. S4. Representative flow cytometry plots showing the gating strategy for T cells isolated from CNS tissues of acute and chronic EAE mice for intracellular cytokine assays and the quantification of individual cell populations. (a–q) Representative flow cytometry plots showing the gating strategy for T cells isolated from the spinal cord of vehicle-treated (a–d) and INI-0602-treated (e–h) EAE mice on dpi 24, and of vehicle-treated (j–m) and INI-0602-treated (n–q) EAE mice on dpi 50. (r,s) Quantitative analysis of IL-17^{-}IFN-γ^{+} (Th1), IL-17^{+}IFN-γ^{-} (Th17), IL-17^{+}IFN-γ^{+} (Th17/Th1), and FoxP3^{+} cell percentages in CD4^{+} T cells from the spinal cord of vehicle- and INI-0602-treated EAE mice on dpi 24 (r) and 50 (s). All data are presented as means ± SEM (n = 3–4 mice per group). Significant differences were determined using unpaired t-tests. *$P < 0.05$; **$P < 0.01$.

SI Figure S5

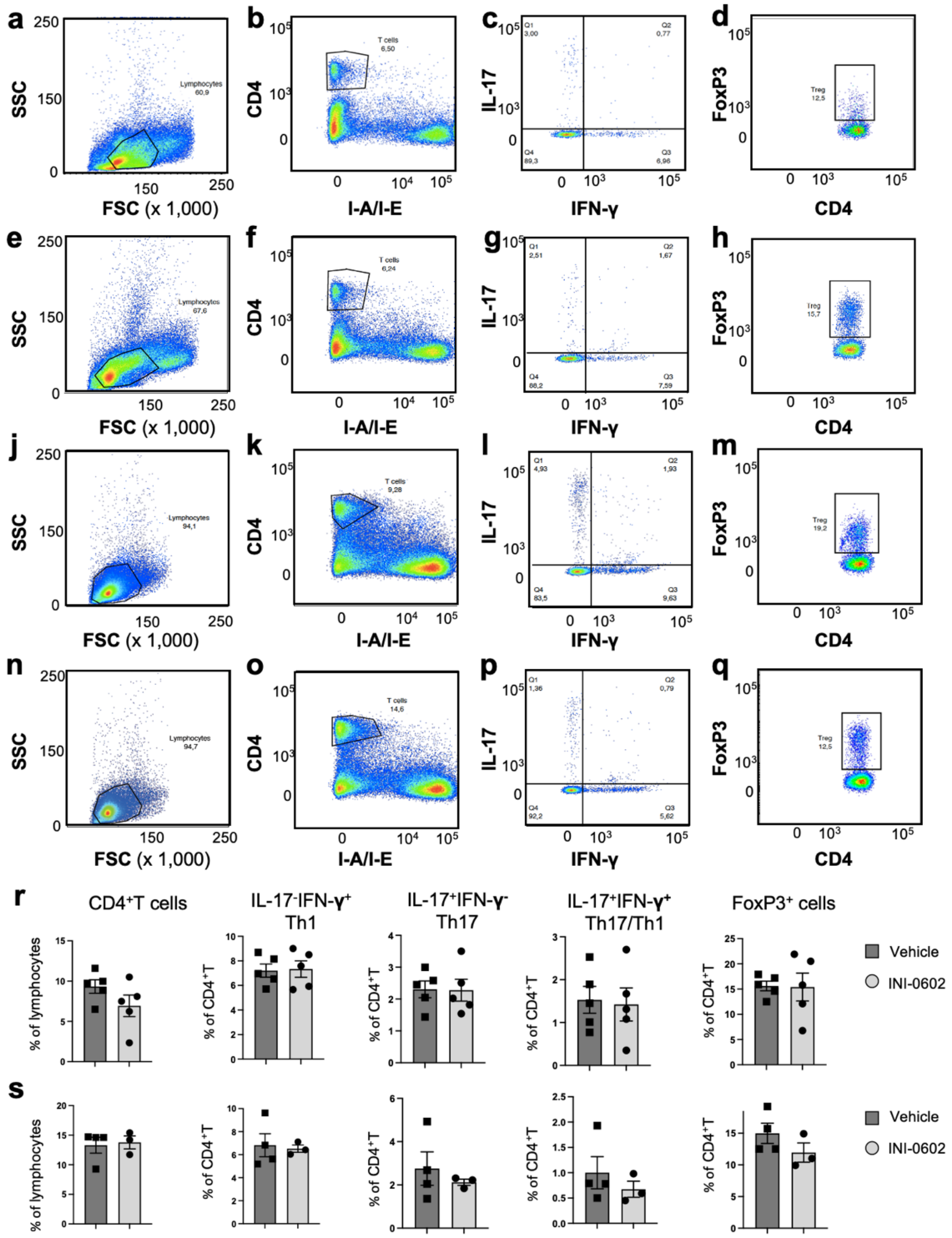
a
b
c
d
e
f
g
h
j
k
l
m
n
o
p
q
r
s
SSC
FSC (x 1,000)
Lymphocytes
CD4
I-A/I-E
T cells
IL-17
IFN-γ
FoxP3
Treg
CD4+T cells
IL-17-IFN-γ+ Th1
IL-17+IFN-γ- Th17
IL-17+IFN-γ+ Th17/Th1
FoxP3+ cells
% of lymphocytes
% of CD4+T
Vehicle
INI-0602

Supplementary Fig. S5. Representative flow cytometry plots showing the gating strategy for T cells isolated from splenocytes of acute and chronic EAE mice for intracellular cytokine assays and the quantification of individual cell populations. (a–q) Representative flow cytometry plots showing the gating strategy for T cells isolated from splenocytes of vehicle-treated (a–d) and INI-0602-treated (e–h) EAE mice on dpi 24, and of vehicle-treated (j–m) and INI-0602-treated (n–q) EAE mice on dpi 50. (r,s) Quantitative analysis of IL-17^{-}IFN-γ^{+} (Th1), IL-17^{+}IFN-γ^{-} (Th17), IL-17^{+}IFN-γ^{+} (Th17/Th1), and FoxP3^{+} cell percentages in CD4^{+} T cells from splenocytes of vehicle- and INI-0602-treated EAE mice on dpi 24 (r) and 50 (s). All data are presented as means ± SEM (n = 3–4 mice per group). Significant differences were determined using unpaired t-tests.

SI Figure S6

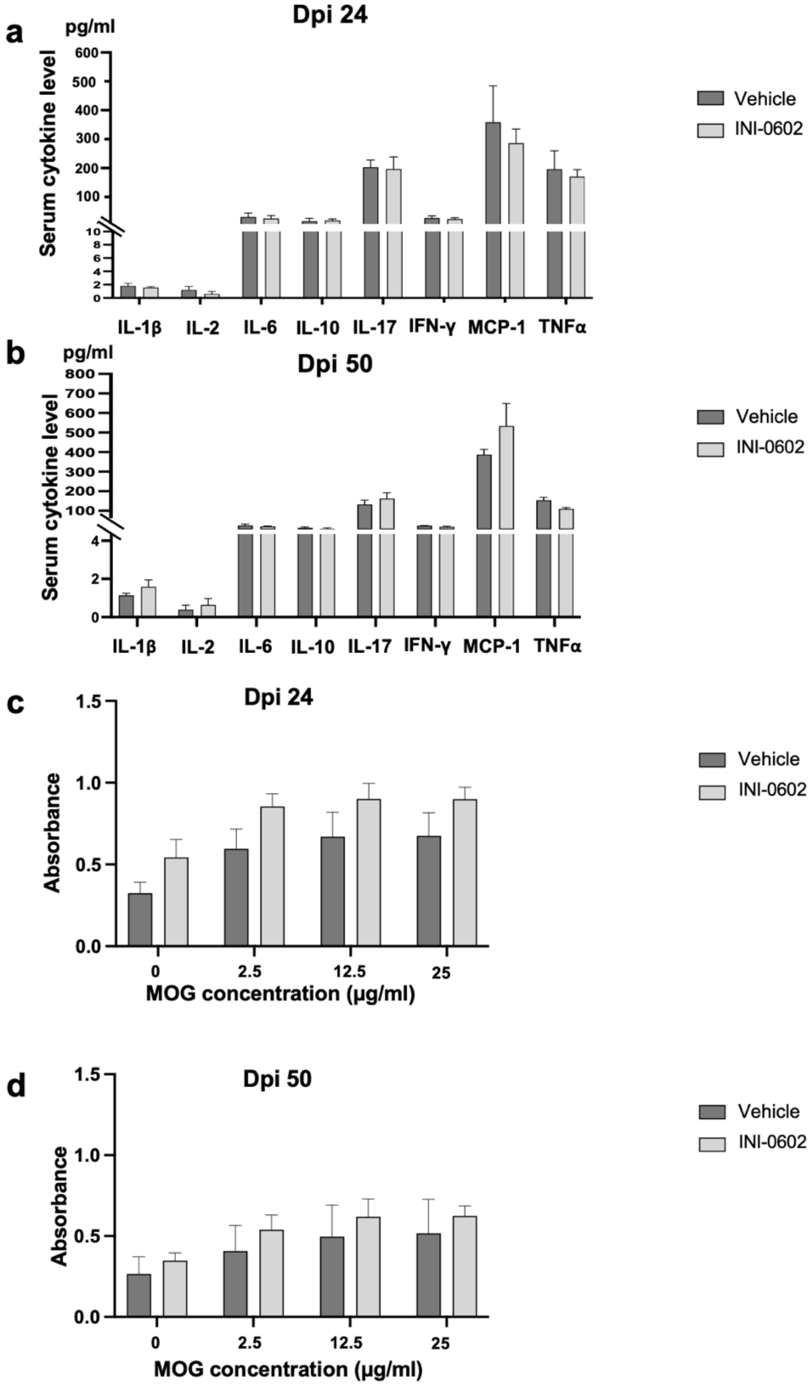
a
Dpi 24
pg/ml
Serum cytokine level
IL-1β
IL-2
IL-6
IL-10
IL-17
IFN-γ
MCP-1
TNFα
Vehicle
INI-0602
b
Dpi 50
pg/ml
Serum cytokine level
c
Dpi 24
Absorbance
MOG concentration (µg/ml)
d
Dpi 50
Absorbance
MOG concentration (µg/ml)

Supplementary Fig. S6. Serum cytokine/chemokine levels and MOG-specific T cell responses in vehicle- and INI-0602-treated EAE mice on dpi 24 (acute) and 50 (chronic). (a,b) Serum was collected from the right atrium of EAE mice at dpi 24 (a) and 50 (b) and was analyzed using a multiplex mouse cytokine assay kit. Each bar represents the mean cytokine level ± SEM. Significant differences were determined using unpaired *t*-tests. (c,d) MOG-specific T cell responses in INI-0602- and vehicle-treated mice. Splenic T cell proliferation was assayed using BrdU incorporation at different MOG concentrations (0, 2.5, 12.5, and 25 μg/ml) in INI-0602- and vehicle-treated mice (n = 3 per group) at dpi 24 (c) and 50 (d). All data are shown as means ± SEM. Significant differences were determined using two-way ANOVA.

SI Figure S7

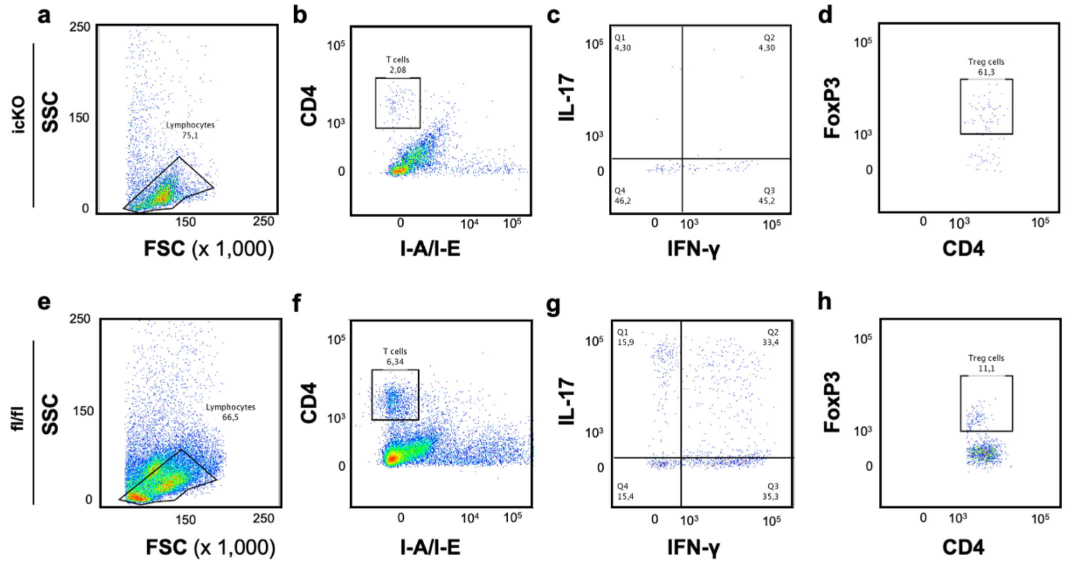
a
b
c
d
e
f
g
h
icKO
fl/fl
SSC
FSC (x 1,000)
Lymphocytes
75,1
Lymphocytes
66,5
CD4
I-A/I-E
T cells
2,08
T cells
6,34
IL-17
IFN-γ
Q1
4,30
Q2
4,30
Q4
46,2
Q3
45,2
Q1
15,9
Q2
33,4
Q4
15,4
Q3
35,3
FoxP3
Treg cells
61,3
Treg cells
11,1

Supplementary Fig. S7. (a–h) Representative flow cytometry plots showing the gating strategy for T cells isolated from the spinal cord of peak (dpi 17) *GLAST*$^{+}$ *Cx43* icKO or *fl/fl* EAE mice for intracellular cytokine assays and the quantification of individual cell populations.

SI Figure S8

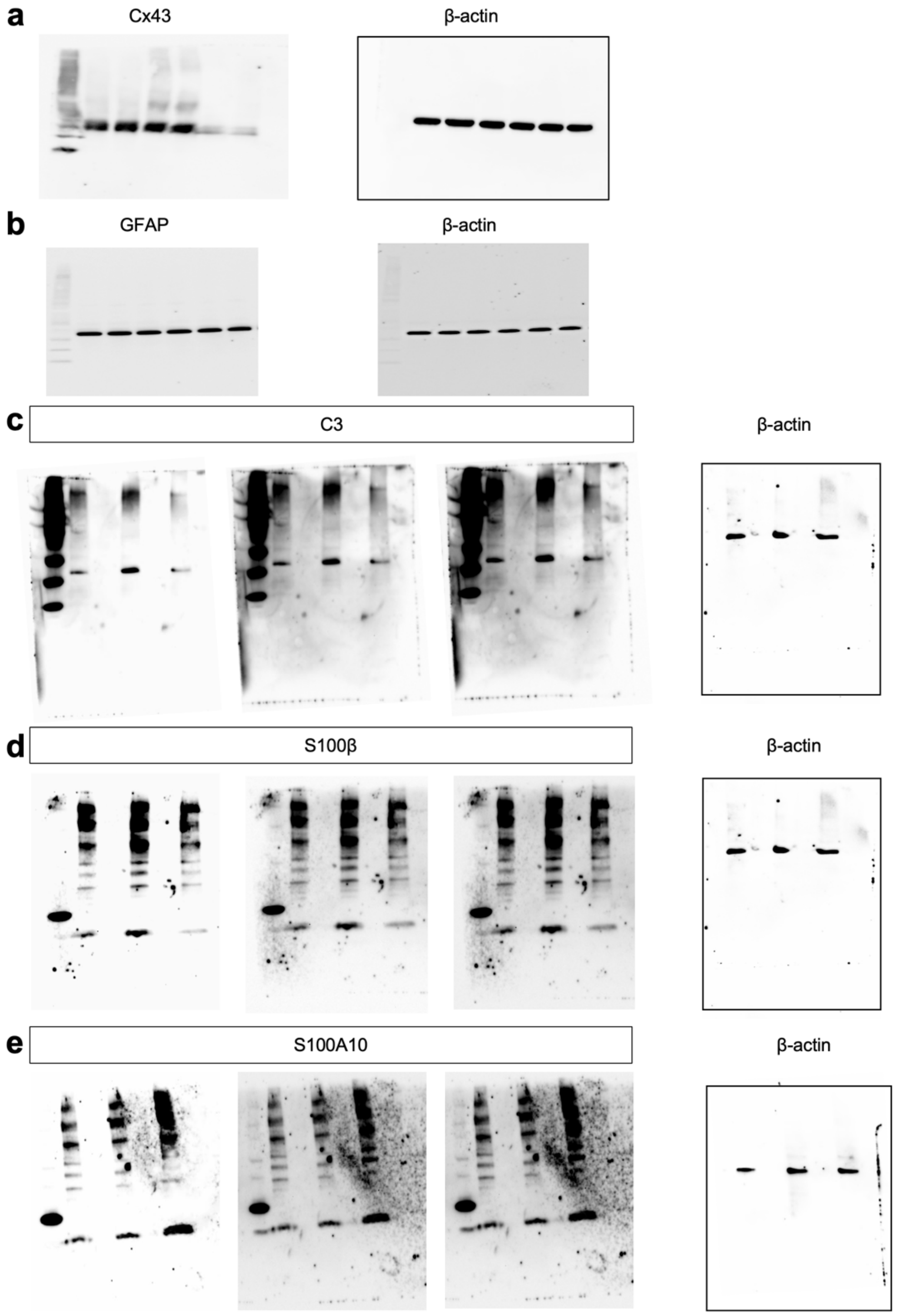
a
Cx43
β-actin
b
GFAP
β-actin
c
C3
β-actin
d
S100β
β-actin
e
S100A10
β-actin

Supplementary Fig. S8. Original blots are presented including multiple exposure images.

SI Figure S9

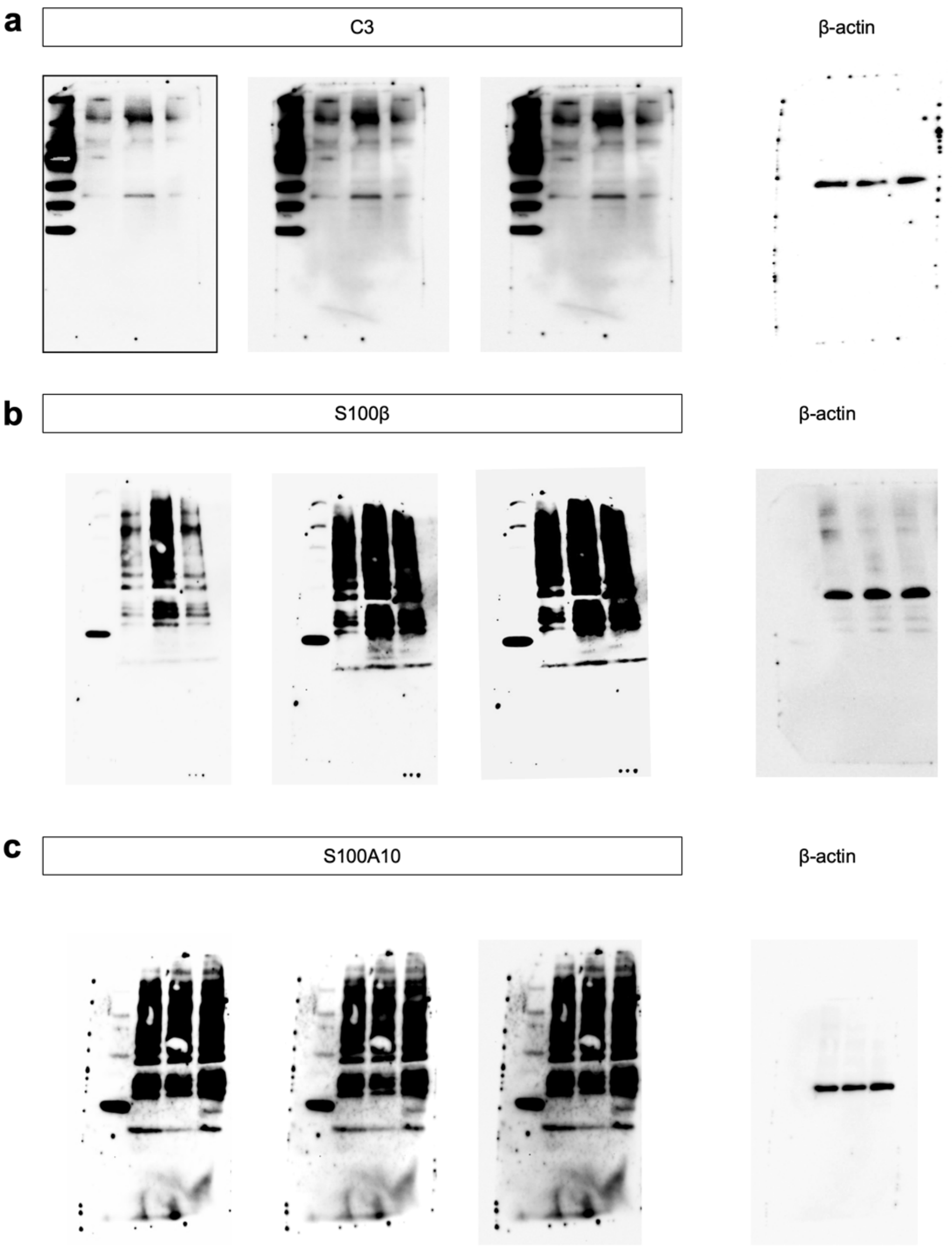

Supplementary Fig. S9. Original blots are presented including multiple exposure images.

SI Table: Antibodies used in this study

Antigen	Clone	Type	Dilution	Incubation Temperature/ Time	Source	Application
**Iba-1**	Polyclonal	Rabbit	1: 1000	4°C Overnight	FUJIFILM Wako	IHC
**C3**	Monoclonal (11H9)	Rat	1: 50	4°C Overnight	HycultBiotech	IHC
**GFAP**	Monoclonal (2E1.E9)	Mouse	1: 1000	4°C Overnight	STEMCELL	IHC
**S100Beta**	Monoclonal (EP1576Y)	Rabbit	1:500	4°C Overnight	Abcam	IHC
**Connexin 43/ GJA1 Antibody**	Polyclonal	Rabbit	1: 400	4°C Overnight	Abcam	IHC
**CD3**	Monoclonal (SP7)	Rabbit	1: 100	4°C Overnight	Abcam	IHC
**F4/80**	Monoclonal (CI:A3-1)	Rat	1: 100	4°C Overnight	Abcam	IHC
**MBP**	Monoclonal (F-6)	Mouse	1: 200	RT Overnight	Santa cruz biotechnology	IHC
**S100A10**	Polyclonal	Goat	1: 100	4°C Overnight	R&D Systems	IHC
**EAAT1**	Monoclonal (EPR12686)	Rabbit	1: 400	4°C Overnight	Abcam	IHC
**EAAT2**	Monoclonal (EPR19798)	Rabbit	1: 400	4°C Overnight	Abcam	IHC

❖ **Buffers: 10% BSA in TBS-Tween 20 0.1%.**

Antigen	Clone	Type	Dilution	Incubation Temperature/ Time	Source	Application
**CD4**	RM4-5, FITC-conjugated	Rat / IgG2a	1: 30	4°C 30 min	Biolegend	FCM
**I-A/I-E**	M5/114.15.2, PE/Cy7 conjugated	Rat	1: 30	4°C 30 min	Biolegend	FCM
**IL-17A**	TC11-18H10.1 APC conjugated	Rat IgG1	1: 30	4°C 30 min	Biolegend	FCM
**IFN-γ**	XMG1.2, PerCP-Cyanine5.5 conjugated	Rat / IgG1	1: 30	4°C 30 min	Invitrogen	FCM
**Foxp3**	FJK-16s, PE conjugated	Rat / IgG2a	1: 15	4°C 30 min	Invitrogen	FCM
